# Reasons for Utilizing Telemedicine during and after the COVID-19 Pandemic: An Internet-Based International Study

**DOI:** 10.3390/jcm10235519

**Published:** 2021-11-25

**Authors:** Arriel Benis, Maxim Banker, David Pinkasovich, Mark Kirin, Bat-el Yoshai, Raquel Benchoam-Ravid, Shai Ashkenazi, Abraham Seidmann

**Affiliations:** 1 Faculty of Industrial Engineering and Technology Management, Holon Institute of Technology, Holon 5810201, Israel; bmaxim1989@gmail.com (M.B.); dudu0906@gmail.com (D.P.); markkirin89@gmail.com (M.K.); batelyo@gmail.com (B.-e.Y.); 2 Faculty of Digital Technologies in Medicine, Holon Institute of Technology, Holon 5810201, Israel; 3 IMA Group Clinic, Rochester, NY 14610, USA; benchoam.r@gmail.com; 4 Adelson School of Medicine, Ariel University, Ariel 4070000, Israel; shaias@ariel.ac.il; 5 Department of Information Systems, Questrom Business School, Boston University, Boston, MA 02215, USA; avis@bu.edu; 6 Health Analytics and Digital Health, Digital Business Institute, Boston University, Boston, MA 02215, USA

**Keywords:** telemedicine, teleconsultation, social distancing, patient satisfaction, coronavirus, COVID-19, SARS-CoV-2, pandemic, eHealth, health services, health care delivery, internet use

## Abstract

The COVID-19 pandemic challenges healthcare services. Concomitantly, this pandemic had a stimulating effect on technological expansions related to telehealth and telemedicine. We sought to elucidate the principal patients’ reasons for using telemedicine during the COVID-19 pandemic and the propensity to use it thereafter. Our primary objective was to identify the reasons of the survey participants’ disparate attitudes toward the use of telemedicine. We performed an online, multilingual 30-question survey for 14 days during March–April 2021, focusing on the perception and usage of telemedicine and their intent to use it after the pandemic. We analyzed the data to identify the attributes influencing the intent to use telemedicine and built decision trees to highlight the most important related variables. We examined 473 answers: 272 from Israel, 87 from Uruguay, and 114 worldwide. Most participants were women (64.6%), married (63.8%) with 1–2 children (52.9%), and living in urban areas (84.6%). Only a third of the participants intended to continue using telemedicine after the COVID-19 pandemic. Our main findings are that an expected substitution effect, technical proficiency, reduced queueing times, and peer experience are the four major factors in the overall adoption of telemedicine. Specifically, (1) for most participants, the major factor influencing their telemedicine usage is the implicit expectation that such a visit will be a full substitute for an in-person appointment; (2) another factor affecting telemedicine usage by patients is their overall technical proficiency and comfort level in the use of common web-based tools, such as social media, while seeking relevant medical information; (3) time saving as telemedicine can allow for asynchronous communications, thereby reducing physical travel and queuing times at the clinic; and finally (4) some participants have also indicated that telemedicine seems more attractive to them after watching family and friends (peer experience) use it successfully.

## 1. Introduction

In an era of data-driven, customer-centered healthcare practice, the whole health ecosystem must be involved in improving and optimizing the components, processes, and systems supporting access to health services and reducing costs. A key challenge of electronic health (eHealth) [1,2] and telemedicine [3,4,5] is to reduce the load on the physical health system infrastructure, particularly in times of crisis.

The COVID-19 pandemic—with the related social distancing, quarantines, and confinements—still challenges the healthcare services and concomitantly stimulates the technological expansion of eHealth and telemedicine. Telemedicine and remote delivery of healthcare services have been declared as global public health responses to gain control over the virus [6,7,8]. However, many healthcare services across the globe are managed and operated differently, due to the architecture of the healthcare systems, the regulatory environment, and the influence of the local culture [9,10,11]. The disparities among healthcare systems are not new and relate to culture and communication [12]. Thus, shifts in communication and culture are crucial to move telemedicine forward and increase the number of users among both clinicians and patients [13].

The current research is international, with a focus on two countries with developed healthcare systems, Israel and Uruguay, whose relative response rate for highest [14,15]. In 2020, the first had 9,291,000 inhabitants [16] and the second 3,473,727 [17].

The Israeli healthcare system is built around four health management organizations (HMOs) and each citizen is mandatorily affiliated with one of them. Since the early 2000s, the overall Israeli healthcare system uses electronic health records and teleservices [18,19,20,21]. Namely, the healthcare infrastructure in Israel was ready to move forward with telemedicine even when the regulations were not fully adapted [8]. During the first waves of the COVID-19 pandemic, the whole healthcare system was overloaded, and visits to clinics and hospitals were restricted. This situation marked a turning point in the popularization of telemedicine. Remote health diagnosis and monitoring tools [22] were used to minimize physical and social contacts with patients with COVID-19 and to prioritize care to patients with chronic and/or multiple comorbidities [23,24,25]. Patient engagement with telemedicine was triggered by its use by healthcare providers. The first COVID-19 lockdown in Israel induced a substantial increase in the number of primary care pediatricians using this technology [26]. Understanding the healthcare customers and providers usages and expectations of telemedicine may also significantly increase their acceptance [27].

The Uruguayan healthcare is organized around two main systems. A large proportion of Uruguay’s population is affiliated with a private system that consists of paying membership in a private hospital that allows the member to receive services from this hospital. On the other hand, the public healthcare system is accessible to any Uruguayan citizen [28]. Uruguay’s parliament established a legal framework for telemedicine at the beginning of the pandemic [29] that allows healthcare providers to manage appointments for care provision by video calls [30].

Across the world, infrastructure, professional training, laws and regulations, and cultural and ethical gaps all present challenges to both practitioners and patients in using telemedicine [7,8,31].

We sought to elucidate the reasons for using telemedicine during the COVID-19 pandemic and the propensity to use it thereafter. Our primary objective was to identify the reasons of the survey participants’ disparate attitudes toward the use of telemedicine.

This cross-sectional, web-based survey research was led by three hypotheses suggesting that the patients’ use of telemedicine during and after the COVID-19 pandemic can be affected by:

- Prior deployment of telemedicine services by healthcare providers;
- A limited number of socio-demographic variables;
- Specific personal reasons (family influence, previous experience, and location).

## 2. Materials and Methods

### 2.1. Participants

From 23 March to 6 April 2021 (between the second and third COVID-19 pandemic waves), we performed an online, multi-center, multi-lingual, cross-sectional survey about the perception and use of telemedicine by healthcare customers (Supplementary Materials Table S1). The survey was administered in English, Hebrew, Spanish, Russian, French, and Arabic [32,33,34]. The survey was built in and performed over Microsoft Forms and hosted by the principal investigator’s institution. Invitations to take the survey were posted by the research team on social media platforms (Facebook (Facebook Inc., Menlo Park, CA, USA), Twitter (Twitter Inc., San Francisco, CA, USA), and LinkedIn (Microsoft Corp., Sunnyvale, CA, USA), without any paid recruitment or advertising services. The survey link was also sent via email to personal and professional contact lists. Some participants shared the survey address broadly as well. Each of these communication channels has its characteristics and population target [35], which also influences the recruitment approach. The response rate was estimated to be 1.96% (491/25,000, considering estimated invitation reach of 18,000 on Facebook, 2000 on Twitter, 2000 on LinkedIn, and 3000 by email). This response rate is considered a moderate one [35].

### 2.2. Data Preparation

All the questions were mandatory except those about country and city of residence and the names of the participants’ healthcare management organizations. The answer set inclusion criteria were declaring a country of residence and completing the comprehensive questionnaire in more than three and less than 30 min.

The manuscript adheres to reporting standards, including the Checklist for Reporting Results of Internet E-Surveys (CHERRIES) [36], Strengthening the Reporting of Observational Studies in Epidemiology (STROBE) [37], and Transparent Reporting of a Multivariable Prediction Model for Individual Prognosis or Diagnosis (TRIPOD) guidelines for reporting observational studies [38]. The computational methodology is reported in the AIMe registry for artificial intelligence in biomedical research [39].

### 2.3. Statistical Analysis

The comprehensive data collected in the context of this research were categorical. Before starting the statistical analysis process, the answers collected in the various languages were aligned. Then, we reformulated part of the data: The answers to multiple-choice questions with a five-point Likert scale (“completely disagree”, “somewhat disagree”, “neutral/no opinion”, “somewhat agree”, and “completely agree”) were aggregated using a three-point scale (“disagree”, “neutral”, “agree”). 

Cronbach’s α was used to measure the internal reliability of the Likert-scale questions related to the reasons for and satisfaction from using telemedicine (α = 0.81). We stratified the survey data by considering the answers to the question “Will you use telemedicine after the COVID-19 pandemic?” and the participant’s country of residence (i.e., Israel, Uruguay, and the rest of the world).

The distribution of the responses is presented as numbers and percentages. Chi-squared tests and Fisher’s exact tests were used to compare the variables.

We then performed a multivariate analysis with decision trees [40]. We built three decision trees using the questions with a significant statistical test value by taking into account the participants that declared their intent to use or not use telemedicine in the future. Moreover, all the questions about prior intentions were not taken into consideration. Decision trees are an easy-to-understand model comprising a set of cascading questions overcoming Simpson’s paradox [41] by disclosing nonlinear interactions between predictors. This allows a better comprehension of the main variables involved in the intended use of telemedicine after the COVID-19 pandemic [42].

The data analysis was performed with R and the “psych” [43] package for computing the answers’ internal consistency for the matrix multi-point scale questions, the “compareGroups” package [44] for statistical computations, and the “rpart” package [45] for the decision-tree processing. In the overall analysis, statistical significance was considered as a two-sided *p* ≤ 0.05.

## 3. Results

We collected 486 answer sets. After applying the exclusion criteria (one answered in less than three minutes, and 12 answered in more than 30 min), 473 answer sets were included in the analysis. Completing the survey questionnaire took a median time of 8 min and 16 s (IQR (6 min and 1 s; 11 min and 23 s)).

### 3.1. Socio-Demographic Characteristics

The participants were mainly from Israel (272/437, 57.5%) (Appendix A Table A1), Uruguay (87/437, 18.4%) (Appendix A Table A2), and the rest from several other countries (Supplementary Materials Table S2) (114/473, 24.1%) (Appendix A Table A3). Most of the participants were women (305/473, 64.6%; 188/272, 69.4%, in Israel; 75/87, 86.2% in Uruguay; and 117/201, 58.2%, elsewhere); married (302/473, 63.8%; 205/272, 75.4%, in Israel; 39/87, 44.8%, in Uruguay; and 97/201, 48.3%, elsewhere) with 1–2 children (250/473, 52.9%; 158/272, 58.1%, in Israel; 54/87, 62.1%, in Uruguay; and 92/201, 45.8%, elsewhere). Moreover, 63.2% (72/114) of the non-Israeli and non-Uruguayan participants reported that they did not have children.

### 3.2. Prior Use of Online Medical and Non-Medical Services

In Israel, participants who used telemedicine before the pandemic were likely to intend to continue to use it after the pandemic (59.7%, 83/139) (Appendix A Table A4). This contrasts with the Uruguayan responders and those in the rest of the world, where a majority that used telemedicine before the COVID-19 outbreak did not plan to continue to use it or were still undecided (Uruguay, 58.1%, 12/21; elsewhere in the world: 53.6%, 15/28) (Appendix A Table A5 and Table A6). The proportion of survey participants who were undecided about continuing to use telemedicine after the pandemic was relatively high in all three groups (29.0% or 79/272 in Israel, 28.7% or 25/87 in Uruguay, and 41.2% or 47/114 elsewhere).

One important finding was no significant link, in Israel and Uruguay, between using online services (e.g., online shopping), and the willingness to use telemedicine after the pandemic (*p* = 0.225 and *p* = 1.000, respectively). However, this link was significant for the participants in the other countries (*p* < 0.0001); they have all used online services in the past, but many were still undecided about using telemedicine in the future (41.2%, 47/114).

It is intriguing to notice that the use of non-medical online services allowed non-Uruguayan participants to feel more comfortable when using telemedicine (*p* < 0.0001). Additionally, among Israelis, being a frequent social media user was related to the intent to use telemedicine (53.6%, 113/211, *p* < 0.001). However, social media use and information searches before visiting a physician were not significantly related for the Uruguayan participants (*p* > 0.249). Elsewhere, the less the participants used social media the less was their intention to use telemedicine after the pandemic (56.4%, 22/39, *p* < 0.0001). This variation among participants can mean that cultural factors have a broad influence on consumption of healthcare services [35,46].

### 3.3. Health Services Consumption

#### 3.3.1. Consumers’ Habits

The frequency of the responders’ use of online health services was positively related with the intent to use telemedicine after the pandemic (Tables S3–S5), in Israel (*p* < 0.001), in Uruguay (*p* = 0.114) and elsewhere (*p* = 0.064). We noticed that fewer of those who never or seldom used health services (never or 1–4 times a year) intended to use telemedicine (Israel: 51.1.6%, 71/139; Uruguay: 66.7%, 15/21; and elsewhere: 75.0%, 21/28). When we looked at the communication channels preferred for communicating with healthcare practitioners, with no face-to-face meeting, a significant difference existed between the participants interested in continuing to use telemedicine and the others for the Israeli participants (*p* = 0.007). The participants mostly preferred phone calls (34.2%, 93/272). This channel was mainly used by those who did not intend to use or were not sure about using telemedicine in the future (50.0%, 27/54). Moreover, the Israeli participants seemed to make much more use of the message “Write to doctor” feature proposed by the HMO online platforms (30.5%, 83/272) than the other participants (3.98%, 8/201), perhaps as their healthcare providers did not offer this option. The use of the messaging system also highlights the importance of asynchronous telemedicine in daily practice [18], as we discuss below. Furthermore, it is important to emphasize that both in Uruguay (*p* = 0.667) and elsewhere in the world (*p* = 0.872) there were no particular preferences for using one of the telemedicine tools over not using telemedicine.

#### 3.3.2. Non-Queue Requests

Many interactions with healthcare providers can occur without needing to have synchronous contact. This kind of interaction is known as a “non-queue request” (NQR), which is an asynchronous channel of communication between patients and healthcare providers [18,47]. NQRs usually relate to administrative actions, such as:

- scheduling an appointment with a healthcare practitioner;
- asking for a prescription or prescription renewal;
- requesting a sickness leave/certificate of absence;
- asking for a referral to a specialist;
- getting laboratory or imaging test results;
- purchasing drugs at an online accredited pharmacy.

Israeli HMOs allow their members to submit an NQR by a face-to-face visit at a clinic, by calling a clinic or call center, or via the HMO’s website or application. The Israeli survey participants that frequently employed NQRs mostly intended to use telemedicine after the COVID-19 pandemic. Indeed, 79.0% (215/272) of the responders currently requested prescriptions online, of whom 55.8% (120/215) planned to continue use telemedicine (*p* = 0.001). Similarly, 56.2% (153/272) schedule healthcare appointments online, of whom 61.4% (94/153) intend to use telemedicine in the future (*p* < 0.001). The same behavior related to requesting sickness leave or a certificate of absence (58.5%, 159/272, *p* = 0.006). It is important to point out that, in Israel, online requests for prescription renewal are allowed only for those receiving a long-term treatment and who consult the relevant physician in person at least once a year. Moreover, the physician can decline the request and ask to meet the patient (in person or online).

The Uruguayan participants also currently made use of the same online services, but they lacked the intent to continue to use telemedicine. Many of these respondents also consulted test results online (73.6%, 64/87, *p* = 0.112) but 70.3% (45/64) of them were still undecided about telemedicine or declared that they will not use it later. Likewise, 57.5% (50/87) and 56.3% (49/87) asked for a prescription by going to the clinic or scheduled a consultation online, but in both cases those who were undecided or who would not use telemedicine in the future were a majority (66.0%, 33/50, *p* = 0.019, and 65.3%, 32/49, *p* = 0.030, respectively).

The participants located elsewhere in the world gave mixed answers. Many scheduled appointments online (64.9%, 74/114). However, there was no significant difference in the intent to use telemedicine. Nevertheless, online services that are less used (perhaps due to low deployment in the respondents’ country) showed significant differences regarding the intent to continue using telemedicine in the future. For example, only around 16.0% of the participants requested a referral to a specialist or seeking a specialist’s opinion via an NQR, but more than half expected to continue telemedicine usage, *p* < 0.018.

Additionally, most of the participants using telemedicine intended to use it in the future (71.4%, 15/21, and 46.4%, 13/28, respectively). This shows that using a service that has an impact on the quality of life (by eliminating the need to go to the doctor) reinforces the intent to continue to use telemedicine after the pandemic.

Overall, about half of the participants consulted the results of laboratory or imaging tests (such as blood tests, smears, or X-rays) online (49.9%, 236/473), and less than half of those (48.7%, 115/236, *p* < 0.001) had a positive disposition to use telemedicine in the future. 

#### 3.3.3. Teleconsultation and Biometry

Involving telebiometry in remote consultations is not something new [48,49,50], but its widespread use began a few years ago. The number of participants reporting having a consultation with a health practitioner that involved telebiometry was meager, and most of those were Israelis. Indeed, the Israeli HMOs allow their members to buy and use telehealth devices such as Tyto. Using these tools looks to be linked to a greater likelihood of using telemedicine in the future (83.3%, 15/18, *p* = 0.001).

### 3.4. Participants’ Reasons for Using Telemedicine during and after the COVID-19 Pandemic

Several reasons related to the intend to use or not use telemedicine in the post-pandemic era were explored.

#### 3.4.1. Family, Friends, and Others

Having close family members using telemedicine seems to have a highly significant impact on the intent to use telemedicine services in the future. Of the participants having some family members already using telemedicine, 69.7% (131/188) intended to use telemedicine (*p* < 0.001) and 51.7% (78/151) had not decided yet. In Israel, these values were higher: 70.5% (98/139) and 58.2% (46/79), respectively (*p* < 0.001). Additionally, for Israeli participants, knowing friends and coworkers who used telemedicine was significantly related to the intent to use telemedicine, at 60.3% (70/116) and 68.8% (53/77), respectively, (*p* < 0.001).

In Uruguay, the results were similar: 51.7% (45/87) and 46.0% (49/87) of the participants had family members and friends using telemedicine, respectively; a high proportion of these participants intend to use telemedicine after the pandemic or had not decided yet (73.3%, 33/45, *p* < 0.001, and 60.0%, 24/40, *p* = 0.225).

In other countries, a lower association was noted between the intent to use telemedicine in the future and its use by family members (having a positive intent: 36.2%, 17/47; being undecided: 31.2%, 15/47, *p* = 0.045), and by friends (30.7%, 35/87, *p* = 0.266) and coworkers (20.2%, 23/87, *p* = 0.220). 

#### 3.4.2. Saving Time

The reasoning of saving time by telemedicine use was another discriminant (*p* < 0.001) between the Israeli participants and the others. Of the 72.4% (197/272) of the Israelis who thought that using telemedicine saved time, 58.4% (115/197) intend to continue using this healthcare channel in the future, higher than in those who did not appreciate the time saving (*p* < 0.001). Contrasting with Israel, relatively few of the Uruguayan participants were motivated to use telemedicine to save time (16.1%, 14/87), of whom 57.1% (8/14, *p* = 0.003) had a positive willingness regarding future use. 

The results among participants from other locations were different; telemedicine was considered as a “time saver” and an “opportunity to get a medical answer anytime and anywhere” by most individuals (67.5%, 77/114), but only 31.2% (24/77) intended to use telemedicine in the future.

Few participants (5.92%, 28/473) reported using telemedicine in situations of a medical emergency, and this kind of use did not show a significant relation to the intent to use telemedicine in the future (*p* > 0.173).

### 3.5. Technology and Communication as Users’ Satisfaction Triggers

As technological and communication issues can be important barriers against using telemedicine, they were examined in the survey (Tables S6–S8).

#### 3.5.1. Technological Issues

Differences existed among the locations of the participants regarding the factors that bothered them during online consultations. For the Israeli participants, three factors looked problematic. The most critical point was related to the inability of the physician using telemedicine to perform a basic physical examination (48.2%, 131/272, *p* = 0.044). Of those who were concerned with this limitation, the majority still intended to continue telemedicine use (56.5%, 74/131). For 18.4% (50/272), interruption of consultations without the possibility of renewing the call was a problem, although most of them (68.0%, 34/50) intend to continue consulting a doctor in this way. A small percentage of the participants located elsewhere around the globe answered that this type of interruption was bothersome (9.75%, 11/114, *p* < 0.0001), but 81.2% (9/11) of those respondents intend to continue using telemedicine. The Uruguayan participants did not report a significant difference in the issues encountered during teleconsultation. However, these participants did not express fear of receiving an answer from a non-specialist doctor during a written exchange (*p* < 0.0001) or that the consultation did not take place (*p* < 0.0001). Many participants were concerned by the physician’s inability to perform a basic physical examination (60.9%, 53/87) and that he would not understand the patient’s feelings and problems (41.4%, 36/87).

#### 3.5.2. Communication Issues

Israel is a nation of immigrants, with a blend of different cultures; Hebrew is spoken by natives and also by old or new immigrants to varying levels of proficiency. We thus intentionally explicitly asked a dual-matter question related both to language proficiency and service perception. We received responses that the “fear of being misunderstood and that the treatment will be of less quality compared to a face-to-face meeting” was an important factor reducing the participant’s willingness to use telemedicine after the pandemic. Indeed, for 27.2% (74/272) of the Israeli participants, 23.0% (20/87) of the Uruguay ones, and 21.1% (24/114) of the participants located elsewhere, this concern induced a relatively high hesitancy (undecided or negative intent) about using telemedicine (Israel, 59.5%, 44/74, *p* = 0.054; Uruguay, 45.0%, 9/20, *p* = 1.000; and elsewhere, 87.5%, 21/24, *p* = 0.055). It is important to highlight that the Israeli healthcare systems try to provide multilingual services, so language may not actually be a barrier to online services in the future [51,52,53]. 

We noticed that the less that Israeli participants experienced a need for a face-to-face consultation after an online one, the higher was their willingness to use telemedicine (31.6%, 81/272, *p* < 0.001). Among Uruguayan participants in the survey, the greater the perceived need for an additional face-to-face meeting with the healthcare practitioners (37.9%, 33/87), the lower was the intent to use telemedicine in the future (*p* ≤ 0.051). Elsewhere in the world, the fear of not being understood was not significantly different between the potential telemedicine users and others (*p* = 1.000), but again, the greater the perceived need for follow-up face-to-face consultation, the lower was the intent to use telemedicine in the future (*p* = 0.013).

#### 3.5.3. User Satisfaction and the Impact of the Pandemic

The perception of telemedicine has changed during the pandemic, with an increase among participants in Israel (48.9%, 133/272) and in Uruguay (39.1%, 34/87). Specifically, the majority (56.8%, 79/139) of Israeli participants intended to use telemedicine, although moderate satisfaction of its use was expressed by only 33.5% (91/272) of the whole group and by only 40.3% (56/139) of those intending to continue using telemedicine. Additionally, the Israeli participants who believed that telemedicine use would not progress at the expense of traditional face-to-face practice in clinics had a smaller intent to use telemedicine in the future (44.1%, 120/272). These values are significant (*p* < 0.001). The answers of the Uruguayans were actually similar; most of those who declared that their perception of telemedicine had changed would use telemedicine in the future (71.4%, 15/21). Additionally, the more the participants were satisfied, the higher was their intent to use telemedicine (57.1%, 12/21). According to the Uruguayan answers, no major impact of telemedicine on the classical practice will take place (87.0% 67/87, *p* < 0.001).

User satisfaction was a crucial factor for expansion of telemedicine use and popularity. The survey participants who were interested in using telemedicine in the future were significantly more satisfied users of the service (Israel: 61.5%, 56/91; Uruguay: 38.7%, 12/31; and elsewhere: 47.4%, 18/38) than those not interested (*p* < 0.008). Moreover, the proportion of participants who were neutral regarding their intent to use telemedicine was encouraging when we looked at those who were at least “somewhat satisfied” with telemedicine already and thus might be persuaded to use it in the future (Israel: 27.5%, 25/91; Uruguay: 41.2%, 13/31; and elsewhere: 39.5%, 15/38).

A large proportion of the survey participants were neither satisfied nor dissatisfied (33.4%, 158/473), however, and 45.6% (72/158) of those had a positive willingness to continue using telemedicine in the future. These values may reflect the complexities of the current circumstances for healthcare. On the other hand, many of the participants declaring that they would not use telemedicine after the pandemic had never taken part in an online medical consultation (40.3%, 54/134), and often declared that they were neither satisfied nor dissatisfied (28.4%, 38/134). 

Having helped a senior acquaintance or family member to access telemedicine service also seemed to be related to a greater intent to personally use or continue using telemedicine (*p* < 0.108).

### 3.6. Multivariate Analysis with Decision Trees

Decision trees have been defined as an alternative to logistic regression in epidemiology and public health research. This machine-learning approach fulfills the same goals but with fewer hypotheses and more accurately identifies homogeneous subgroups of individuals by combining a set of characteristics. Moreover, decision trees allow the researcher to develop more accurate profiles of individuals and provide an easy way to interpret the generated results [54,55,56].

**Figure 1:**
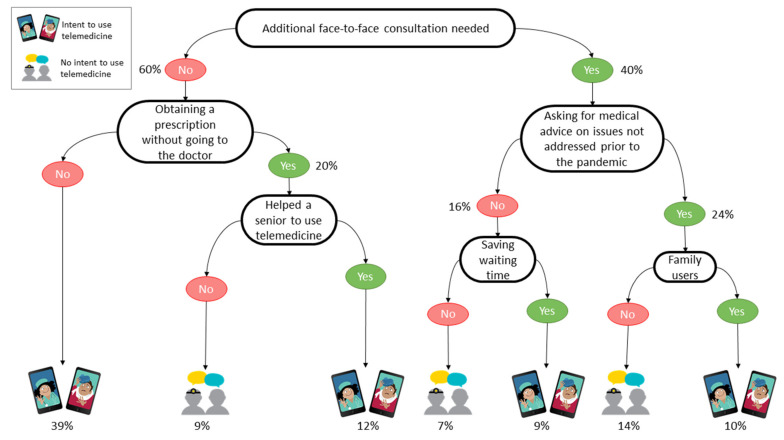
Decision tree of the intent to use telemedicine by Israeli participants.

**Figure 2:**
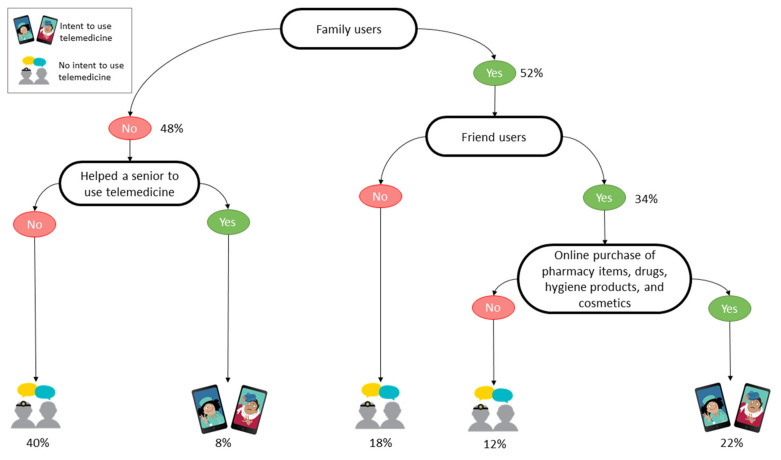
Decision tree of the intent to use telemedicine by Uruguayan participants.

**Figure 3:**
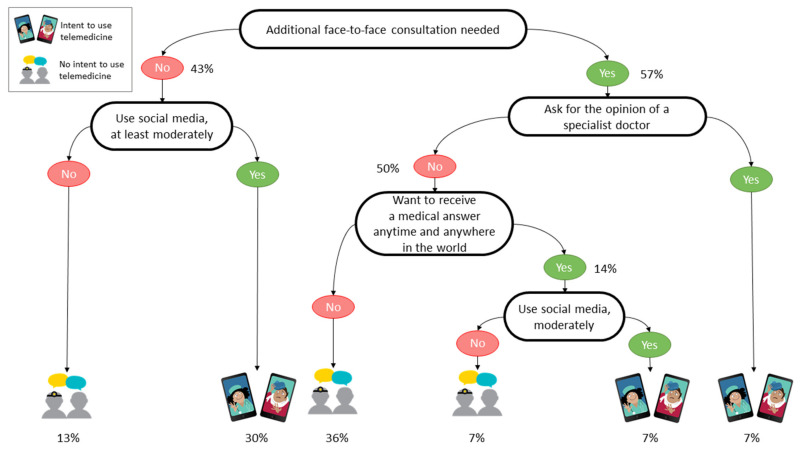
Decision tree of the intent to use telemedicine by the participants located elsewhere in the world.

We built three regression decision trees to support both telemedicine and health communication specialists in targeting their efforts to increase telemedicine use by the public. The decision trees relate, respectively, to the survey’s Israeli participants (Figure 1), the Uruguayan participants (Figure 2), and the participants elsewhere in the world (Figure 3).

The Uruguayan participants (Figure 2) were mainly driven to use telemedicine if first-degree family and friends used it previously, and more particularly when the same participants were using online services to purchase healthcare-related products (22%). In addition, even if close people were not using telemedicine, helping seniors to do so appeared to be a positive trigger (8%). 

The participants located neither in Israel nor in Uruguay (Figure 3) who did not feel the need to have additional face-to-face consultations and who used social media frequently (30%) were more intent on using telemedicine after the pandemic. Moreover, even if they had the need to consult a physician in person, the participants interested in acquiring a specialist opinion online were also potential telemedicine users (7%). Furthermore, the ability to seek medical advice anytime and anywhere was a trigger to use telemedicine for people using social media (7%).

We computed the performance in prediction of the decision trees by having the models built on 66% of the answer sets and then tested the learned models on the remaining 34% and a 10-fold cross-validation, respectively, for the whole answer set, the answers related to Israeli participants, those related to the Uruguayan participants, and those related to the other participants. The decision tree related to the Israeli participants (Figure 1) predicted the intent to use telemedicine with an accuracy of 74.2%. The second model, related to the Uruguayans (Figure 2), had an accuracy of 75.0%, and the last model, related to the participants from elsewhere in the world, predicted the intent to use telemedicine with an accuracy of 65.2% (Figure 3).

## 4. Discussion

### 4.1. Principal Findings

This online survey enabled us to identify seven major axes of reasoning for an intent to use telemedicine after the COVID-19 pandemic.

1. The overall survey results show that about 40% of the participants intend to continue using telemedicine after the COVID-19 pandemic (188/473, 39.7%). A higher proportion of the Israeli than other participants intend to continue using telemedicine services after the pandemic. It is important to point out that telemedicine have been well-established as a part of the HMOs services in Israel for the last decade [57,58], increasingly used as a standard of care since the beginning of the 2000s [59].
2. The major factor influencing the intent to use telemedicine is that a teleconsultation must not require an additional face-to-face consultation in person.
3. Moreover, prior use of online services does not always influence the intent to use telemedicine. However, prior use of non-medical online services does help patients to use and to continue to use telemedicine tools. For Israeli participants, frequent use of social media relates to a higher intent to use telemedicine than it does for those in other countries. Additionally, searching for medical information on the Internet increases the intention to be a telemedicine user.
4. Non-Queue Requests are appreciated by the survey’s participants, and especially by the Israelis. However, it is critical to bear in mind that delivering medical documents raises ethical and legal issues, such as whether to rely on a patient’s online history and whether to renew a prescription without examining the patient [60,61].
5. Telebiometry-based consultation is used in Israel much more than in other countries, perhaps as it is a service delivered in the framework of the HMOs, which have the required technical infrastructure [62,63].
6. Triggers for using telemedicine include being close to people who have used it, the need to feel safe by reducing the number of physical contacts, and the perception that it saves time by reducing unnecessary visits to the provider.
7. A crisis situation such as the COVID-19 pandemic has an influence on perceptions of telemedicine and so points out the need for the healthcare system to be prepared for drastic changes at any time and to be reactive when it is called for.

### 4.2. Strengths and Limitations

An online survey has, by default, a methodological limitation. Indeed, this approach excludes those who are not online. However, the widespread use of the Internet may alleviate such concerns. With 7.68 million Internet users at the beginning of 2021, Israel has an Internet penetration rate of 88.0%; 59.5% of the worldwide population is connected [64,65]. Moreover, a potential risk exists that survey participants will not declare their correct country of residence. However, as with any other survey, one key factor is having high trust in the participants. Additionally, it is challenging to run this kind of survey (disseminated “manually”) without using paid and automated systems that generate very high response rates due to their targeting and potential granting of rewards to each participant. Nevertheless, our survey includes participants from various countries and with different experience of telemedicine, in contrast with research investigating satisfaction with services provided locally [66].

Another limitation of the current research is the difference in the median age of the responding groups: the median age of the Israeli participants is 35–44 years old [67], of the Uruguayans 55–64 [68], and of participants located elsewhere 25–34. This difference in the age groups of the participants can have an impact on the results. For example, the Uruguayan participants seem less interested in using telemedicine than the Israelis, who are much younger. This could be explained by lower adaptation to and acceptance of technology (73–76). However, the younger participants living in other countries also have low intent to be telemedicine users after the pandemic, so the difference in attitude is not merely age-related. Rather, the low intent among these respondents could be related to lower use of healthcare services due to better health [18,69]. Moreover, such differences can partially explain the differences in needs and in intent to use telemedicine [70]. The survey results demonstrate that the intent to use telemedicine depends more on personal perceptions of healthcare needs and the quality of service received, such as technological developments and their added value (e.g., usability of the tools, flexibility for finding medical advice, saving time, and feeling safer through social distancing) than on socio-demographic variables.

As of October 2021, many countries were experiencing their fourth wave of the COVID-19 pandemic, parallel with vaccination campaigns against the disease [71,72] and the implementation of COVID-19 vaccine passports or certificates [73,74]. Even if many individuals adhere to these measures, a non-negligible part of the population shows vaccination hesitancy, which may induce a healthcare system overload. Our study shows that telemedicine will continue to be utilized by a third of the participants, and another third are still undecided. The last third includes those who will not use telemedicine, although they had actually used it during the preceding waves and lockdowns. Additionally, we must underline that a critical trigger for the use of telemedicine during the pandemic and the willingness to employ it is related to prior implementation of the necessary technologies and the degree of their acceptance by the population. Israel has successfully used telemedicine over various communication channels over time, and it seems that Israeli participants’ higher intent to use it in the future can be related to these prior developments and healthcare users’ familiarity with them even though a clear regulation is not defined [75]. In Uruguay, a nationwide integrated healthcare system, through a national e-health platform, is promoted to provide healthcare customers with better access to offline, online, and in-person services [76]. Indeed, during the pandemic, 90% of consultations were conducted by phone, and patient acceptance of telemedicine grew. This approach has been supported by a new regulation to increase telemedicine development [77,78,79]. Uruguay’s healthcare strategy thus has potential to take telemedicine forward by developing appropriate healthcare and legal frameworks [29].

Our findings may have implications in the specific focus on telemedicine-related technology development and policy making. This research mainly focused on relatively small countries (Uruguay, around 3.5 million inhabitants and Israel around 9.2 million), with advanced healthcare systems. Both Israel and Uruguay show that telemedicine gains support due to cultural considerations (e.g., in Israel, saving time, and in Uruguay, seeing that friends and family are also using it) and institutional motivations (e.g., in Israel, the technologies have been implemented by the Health Organization, and in Uruguay, the government established a regulatory framework to increase people’s confidence). Our results can support bigger countries in understanding which way(s) can be used to mature and expand the use of telemedicine, such as improving health communication for reducing fear and increasing the expression of new needs by healthcare users or supporting the improvement of infrastructures and regulations [80].

## 5. Conclusions

### 5.1. Implications for Healthcare Practice

The use of telemedicine outpatient visits has increased dramatically during the COVID-19 pandemic in many countries. Although disparities in access to telemedicine by age and socioeconomic status have been well-documented, evidence is limited as to how these disparities were changed during the pandemic. In Japan, for example, younger individuals increased their use of telemedicine compared to older individuals, although individuals in their 70s also increased their use of telemedicine [81].

Healthcare policymakers should consider telemedicine as a crucial component of the health services ecosystem. The Israeli HMOs identified telemedicine a few years ago as a way to reduce system overload. From non-queue requests to home monitoring of low-risk COVID-19 patients, telemedicine is a part of the care management arsenal that patients, their caregivers, and medical teams are using daily. Therefore, the Israeli participants in this survey are more intent on using telemedicine after the pandemic. Indeed, they were not exposed to the large range of distance-medicine tools only in the emergency situation of the pandemic; rather, the infrastructure was pre-existing and operational [59,82].

Reaching the different healthcare customer profiles and inducing current telemedicine users to continue using it, or persuading others to begin using telemedicine, is critical. It is therefore suggested that health communication specialists and policymakers develop advertising and educational campaigns explaining when and why it is beneficial to use telemedicine. They should target those who are unwilling or ambivalent to telemedicine use, in parallel with additional campaigns that target current telemedicine users as a retention program. It is of prime importance that public health communication with a specific audience employ adapted and personalized language that considers personal opinions and fears [35]. The telehealth and telemedicine adoption rates vary among different populations for several reasons, so these campaigns should address specific social and cultural determinants for each targeted subpopulation [42,83,84].

### 5.2. Current and Potential Future Research Directions

Many countries around the world have developed telemedicine services, but it seems that they have not reached their full potential yet. The COVID-19 pandemic has accelerated changes in consumers’ habits, including their healthcare interactions. Telemedicine is known as an efficient methodology for personal consultations, mainly in primary care, as has been demonstrated during the pandemic. Nevertheless, not all telemedicine channels are equally suitable for all healthcare customers and providers [18,85,86]. The future is going to be integrative and will have to consider the individual’s needs through personalization and flexible support [87,88].

Our cross-sectional and multilingual survey spread over widespread communication channels (social media platforms, emails, and instant messaging services) allowed us to validate our initial hypotheses. Indeed, the use of telemedicine during the pandemic and the intent to use it in the future among the health customers who participated in the survey, from Israel, Uruguay, and elsewhere, are motivated by the combination of several factors, such as prior use of telemedicine services, socio-demographic parameters, and local cultural attitudes.

Our results can help the healthcare industry and policymakers to better understand the factors influencing specific population segments [89]. This understanding can in turn help to improve the development of telemedicine services that better fit the healthcare consumers’ needs and expectations as well as the targeting of communication campaigns aimed at increasing the use of technological tools to facilitate interactions between healthcare consumers and providers [90,91,92].

Future research might consist in running communication campaigns, in various languages when relevant, focusing on saving time or on health safety, over all the communication media and more specifically on social media, by delivering messages encouraging healthcare users to engage with telemedicine tools. Moreover, these campaigns should be targeted by looking at socio-demographics, health services consumption attitudes and, when possible, personal interests in social media profiles [93].

## Data Availability

The data that support the findings of this study are available from the corresponding author (AB) upon reasonable request, which will need to undergo ethical and legal approvals by the institutions of the investigators of the research.

## Supplementary Materials

The following are available online at https://www.mdpi.com/article/10.3390/jcm10235519/s1, Table S1: Survey questionnaire, Table S2: List of the countries of the participants’ not located in Israel or in Uruguay, Table S3: Health services consumption by the Israeli participants in the survey, Table S4: Health services consumption by the Uruguayan participants in the survey, Table S5: Health services consumption by the participants in the survey located elsewhere in the world, Table S6: Technology and communication as satisfaction triggers of the Israeli participants in the survey, Table S7: Technology and communication as satisfaction triggers of the Uruguayan participants in the survey, Table S8: Technology and communication as satisfaction triggers of the participants in the survey located elsewhere in the world.

## Appendix A

**Table A1:** Socio-demographic characteristics of the Israeli participants in the survey by intention to use telemedicine in the future.

Variable	Intention to Use Telemedicine in the Future				
	Overall (n = 272)	Intent (n = 139)	Undecided (n = 79)	No Intent (n = 54)	*p*-Value
What is your age? (n = 271)					<0.001
18–24	9 (3.32%)	3 (2.16%)	6 (7.59%)	0 (0.00%)	
25–34	66 (24.4%)	37 (26.6%)	16 (20.3%)	13 (24.5%)	
35–44	77 (28.4%)	42 (30.2%)	17 (21.5%)	18 (34.0%)	
45–54	68 (25.1%)	36 (25.9%)	21 (26.6%)	11 (20.8%)	
55–64	36 (13.3%)	17 (12.2%)	12 (15.2%)	7 (13.2%)	
≥65	15 (5.54%)	4 (2.88%)	7 (8.86%)	4 (7.55%)	
What is your gender? (n = 271)					0.515
Female	188 (69.4%)	93 (66.9%)	55 (69.6%)	40 (75.5%)	
Male	83 (30.6%)	46 (33.1%)	24 (30.4%)	13 (24.5%)	
What is your marital status? (n = 272)					0.087
Single	36 (13.2%)	15 (10.8%)	15 (19.0%)	6 (11.1%)	
Married	205 (75.4%)	113 (81.3%)	54 (68.4%)	38 (70.4%)	
Divorced	21 (7.72%)	7 (5.04%)	9 (11.4%)	5 (9.26%)	
Widower	6 (2.21%)	2 (1.44%)	1 (1.27%)	3 (5.56%)	
No answer	4 (1.47%)	2 (1.44%)	0 (0.00%)	2 (3.70%)	
How many children do you have? (n = 272)					<0.001
0	51 (18.8%)	22 (15.8%)	19 (24.1%)	10 (18.5%)	
1–2	158 (58.1%)	82 (59.0%)	44 (55.7%)	32 (59.3%)	
3–4	58 (21.3%)	32 (23.0%)	15 (19.0%)	11 (20.4%)	
≥5	5 (1.84%)	3 (2.16%)	1 (1.27%)	1 (1.85%)	
In what sort of area do you live? (n = 272)					0.371
City	249 (91.5%)	126 (90.6%)	71 (89.9%)	52 (96.3%)	
Suburb or periphery	23 (8.46%)	13 (9.35%)	8 (10.1%)	2 (3.70%)	
Language used to take the questionnaire (n = 272)					<0.001
Arabic	0 (0.00%)	0 (0.00%)	0 (0.00%)	0 (0.00%)	
English	0 (0.00%)	0 (0.00%)	0 (0.00%)	0 (0.00%)	
French	2 (0.74%)	1 (0.72%)	0 (0.00%)	1 (1.85%)	
Hebrew	141 (51.8%)	87 (62.6%)	38 (48.1%)	16 (29.6%)	
Russian	128 (47.1%)	51 (36.7%)	40 (50.6%)	37 (68.5%)	
Spanish	1 (0.37%)	0 (0.00%)	0 (0.00%)	0 (1.27%)	

**Table A2:** Socio-demographic characteristics of the Uruguayan participants in the survey by intention to use telemedicine in the future.

Variable	Intention to Use Telemedicine in the Future				
	Overall (n = 87)	Intent (n = 21)	Undecided (n = 25)	No Intent (n = 41)	*p*-Value
What is your age? (n = 87)					0.482
18–24	4 (4.60%)	1 (4.76%)	2 (8.00%)	1 (2.44%)	
25–34	3 (3.45%)	0 (0.00%)	2 (8.00%)	1 (2.44%)	
35–44	4 (4.60%)	2 (9.52%)	0 (0.00%)	2 (4.88%)	
45–54	10 (11.5%)	2 (9.52%)	4 (16.0%)	4 (9.76%)	
55–64	60 (69.0%)	16 (76.2%)	14 (56.0%)	30 (73.2%)	
≥65	6 (6.90%)	0 (0.00%)	3 (12.0%)	3 (7.32%)	
What is your gender? (n = 87)					0.521
Female	75 (86.2%)	17 (81.0%)	21 (84.0%)	37 (90.2%)	
Male	12 (13.8%)	4 (19.0%)	4 (16.0%)	4 (9.76% Correct?)	
What is your marital status? (n = 87)					0.864
Single	13 (14.9%)	2 (9.52%)	4 (16.0%)	7 (17.1%)	
Married	39 (44.8%)	10 (47.6%)	13 (52.0%)	16 (39.0%)	
Divorced	28 (32.2%)	7 (33.3%)	7 (28.0%)	14 (34.1%)	
Widower	4 (4.60%)	2 (9.52%)	0 (0.00%)	2 (4.88%)	
No answer	3 (3.45%)	0 (0.00%)	1 (4.00%)	2 (4.88%)	
How many children do you have? (n = 87)					0.955
0	15 (17.2%)	4 (19.0%)	5 (20.0%)	6 (14.6%)	
1–2	54 (62.1%)	14 (66.7%)	14 (56.0%)	26 (63.4%)	
3–4	17 (19.5%)	3 (14.3%)	6 (24.0%)	8 (19.5%)	
≥5	1 (1.15%)	0 (0.00%)	0 (0.00%)	1 (2.44%)	
In what sort of area do you live? (n = 87)					0.263
City	83 (95.4%)	19 (90.5%)	25 (100%)	39 (95.1%)	
Suburb or periphery	4 (4.60%)	2 (9.52%)	0 (0.00%)	2 (4.88%)	
Language used to take the questionnaire (n = 87)					<0.001
Arabic	0 (0.00%)	0 (0.00%)	0 (0.00%)	0 (0.00%)	
English	0 (0.00%)	0 (0.00%)	0 (0.00%)	0 (0.00%)	
French	0 (0.00%)	0 (0.00%)	0 (0.00%)	0 (0.00%)	
Hebrew	0 (0.00%)	0 (0.00%)	0 (0.00%)	0 (0.00%)	
Russian	0 (0.00%)	0 (0.00%)	0.00(0%)	0 (0.00%)	
Spanish	87 (100%)	21 (100%)	25 (100%)	41 (100%)	

**Table A3:** Socio-demographic characteristics of the participants in the survey located elsewhere in the world by intention to use telemedicine in the future.

Variable	Intention to Use Telemedicine in the Future				
	Overall (n = 114)	Intent (n = 28)	Undecided (n = 47)	No Intent (n = 39)	*p*-Value
What is your age? (n = 114)					<0.001
18–24	39 (34.2%)	5 (17.9%)	19 (40.4%)	15 (38.5%)	
25–34	28 (24.6%)	6 (21.4%)	9 (19.1%)	13 (33.3%)	
35–44	17 (14.9%)	10 (35.7%)	5 (10.6%)	2 (5.13%)	
45–54	14 (12.3%)	3 (10.7%)	7 (14.9%)	4 (10.3%)	
55–64	12 (10.5%)	3 (10.7%)	4 (8.51%)	5 (12.8%)	
≥65	4 (3.51%)	1 (3.57%)	3 (6.38%)	0 (0.00%)	
What is your gender? (n = 114)					0.301
Female	42 (36.8%)	13 (46.4%)	18 (38.3%)	11 (28.2%)	
Male	72 (63.2%)	15 (53.6%)	29 (61.7%)	28 (71.8%)	
What is your marital status? (n = 114)					0.055
Single	50 (43.9%)	7 (25.0%)	22 (46.8%)	21 (53.8%)	
Married	58 (50.9%)	21 (75.0%)	21 (44.7%)	16 (41.0%)	
Divorced	1 (0.88%)	0 (0.00%)	1 (2.13%)	0 (0.00%)	
Widower	5 (4.39%)	0 (0.00%)	3 (6.38%)	2 (5.13%)	
No answer	0 (0.00%)	0 (0.00%)	0 (0.00%)	0 (0.00%)	
How many children do you have? (n = 114)					0.750
0	72 (63.2%)	15 (53.6%)	30 (63.8%)	27 (69.2%)	
1–2	38 (33.3%)	12 (42.9%)	15 (31.9%)	11 (28.2%)	
3–4	4 (3.51%)	1 (3.57%)	2 (4.26%)	1 (2.56%)	
≥5	0 (0.00%)	0 (0.00%)	0 (0.00%)	0 (0.00%)	
In what sort of area do you live? (n = 114)					0.305
City	68 (59.6%)	16 (57.1%)	25 (53.2%)	27 (69.2%)	
Suburb or periphery	46 (40.4%)	12 (42.9%)	22 (46.8%)	12 (30.8%)	
Language used to take the questionnaire (n = 114)					0.367
Arabic	0 (0.00%)	0 (0.00%)	0 (0.00%)	0 (0.00%)	
English	67 (58.8%)	17 (60.7%)	27 (57.4%)	23 (59.0%)	
French	40 (35.1%)	9 (32.1%)	18 (38.3%)	13 (33.3%)	
Hebrew	0 (0.00%)	0 (0.00%)	0 (0.00%)	0 (0.00%)	
Russian	5 (4.39%)	0 (0.00%)	2 (4.26%)	3 (7.69%)	
Spanish	2 (1.75%)	2 (7.14%)	0 (0.00%)	0 (0.00%)	

**Table A4:** Prior use of online medical and non-medical services by the Israeli participants in the survey.

Variable	Intention to Use Telemedicine in the Future				
	Overall (n = 272)	Intent (n = 139)	Undecided (n = 79)	No Intent (n = 54)	*p*-Value
Do you use the following online services? Banking services, Food purchases, Clothing purchases, Electrical product purchases, Hotel reservations, Vacation arrangements (planning, purchases), Health consulting, Government services (n = 272)					0.225
No	5 (1.84%)	1 (0.72%)	2 (2.53%)	2 (3.70%)	
Yes	267 (98.2%)	138 (99.3%)	77 (97.5%)	52 (96.3%)	
Do you use social media (reading, posting) for information and/or advice (not necessarily concerning a medical problem)? (n = 272)					<0.0001
Absolutely not	11 (4.04%)	7 (5.04%)	1 (1.27%)	3 (5.56%)	
A bit	50 (18.4%)	19 (13.7%)	17 (21.5%)	14 (25.9%)	
Moderately	103 (37.9%)	48 (34.5%)	36 (45.6%)	19 (35.2%)	
A lot	76 (27.9%)	44 (31.7%)	18 (22.8%)	14 (25.9%)	
Always	32 (11.8%)	21 (15.1%)	7 (8.86%)	4 (7.41%)	
Do you use online medical services? (n = 272)					<0.0001
No, not interested in using them	16 (5.88%)	2 (1.44%)	4 (5.06%)	10 (18.5%)	
No, but willing to experiment	41 (15.1%)	9 (6.47%)	13 (16.5%)	19 (35.2%)	
Yes, I used them for the first time during the COVID-19 pandemic.	87 (32.0%)	45 (32.4%)	30 (38.0%)	12 (22.2%)	
Yes, I used them before the COVID-19 pandemic.	128 (47.1%)	83 (59.7%)	32 (40.5%)	13 (24.1%)	
Before seeing a doctor, do you use social media to find answers to your question? (n = 272)					<0.0001
Absolutely not	65 (23.9%)	33 (23.7%)	20 (25.3%)	12 (22.2%)	
A bit	110 (40.4%)	54 (38.8%)	29 (36.7%)	27 (50.0%)	
Moderately	58 (21.3%)	30 (21.6%)	20 (25.3%)	8 (14.8%)	
A lot	24 (8.82%)	15 (10.8%)	6 (7.59%)	3 (5.56%)	
Always	15 (5.51%)	7 (5.04%)	4 (5.06%)	4 (7.41%)	
Has your knowledge of using other online services helped you use online medical services? (n = 272)					<0.0001
I have not tried online services.	28 (10.3%)	6 (4.32%)	13 (24.1%)	9 (11.4%)	
Absolutely not	14 (5.15%)	4 (2.88%)	7 (13.0%)	3 (3.80%)	
A bit	57 (21.0%)	22 (15.8%)	10 (18.5%)	25 (31.6%)	
Moderately	69 (25.4%)	39 (28.1%)	14 (25.9%)	16 (20.3%)	
A lot	80 (29.4%)	52 (37.4%)	6 (11.1%)	22 (27.8%)	
Absolutely	24 (8.82%)	16 (11.5%)	4 (7.41%)	4 (5.06%)	

**Table A5:** Prior use of online medical and non-medical services by the Uruguayan participants in the survey.

Variable	Intention to Use Telemedicine in the Future				
	Overall (n = 87)	Intent (n = 219)	Undecided (n = 259)	No Intent (n = 41)	*p*-Value
Do you use the following online services? Banking services, Food purchases, Clothing purchases, Electrical product purchases, Hotel reservations, Vacation arrangements (planning, purchases), Health consulting, Government services (n = 87)					1.000
No	2 (2.30%)	0 (0.00%)	1 (4.00%)	1 (2.44%)	
Yes	85 (97.7%)	21 (100%)	24 (96.0%)	40 (97.6%)	
Do you use social media (reading, posting) for information and/or advice (not necessarily concerning a medical problem)? (n = 87)					0.770
Absolutely not	4 (4.60%)	0 (0.00%)	1 (4.00%)	3 (7.32%)	
A bit	19 (21.8%)	3 (14.3%)	6 (24.0%)	10 (24.4%)	
Moderately	38 (43.7%)	10 (47.6%)	11 (44.0%)	17 (41.5%)	
A lot	18 (20.7%)	7 (33.3%)	5 (20.0%)	6 (14.6%)	
Always	8 (9.20%)	1 (4.76%)	2 (8.00%)	5 (12.2%)	
Do you use online medical services? (n = 87)					0.004
No, not interested in using them	11 (12.6%)	0 (0.00%)	0 (0.00%)	11 (26.8%)	
No, but willing to experiment	13 (14.9%)	3 (14.3%)	4 (16.0%)	6 (14.6%)	
Yes, I used them for the first time during the COVID-19 pandemic.	42 (48.3%)	9 (42.9%)	14 (56.0%)	19 (46.3%)	
Yes, I used them before the COVID-19 pandemic.	21 (24.1%)	9 (42.9%)	7 (28.0%)	5 (12.2%)	
Before seeing a doctor, do you use social media to find answers to your question? (n = 87)					0.446
Absolutely not	32 (36.8%)	8 (38.1%)	6 (24.0%)	18 (43.9%)	
A bit	24 (27.6%)	4 (19.0%)	9 (36.0%)	11 (26.8%)	
Moderately	19 (21.8%)	5 (23.8%)	7 (28.0%)	7 (17.1%)	
A lot	9 (10.3%)	2 (9.52%)	2 (8.00%)	5 (12.2%)	
Always	3 (3.45%)	2 (9.52%)	1 (4.00%)	0 (0.00%)	
Has your knowledge of using other online services helped you use online medical services? (n = 87)					0.249
I have not tried online services.	9 (10.3%)	0 (0.00%)	7 (17.1%)	2 (8.00%)	
Absolutely not	14 (16.1%)	1 (4.76%)	9 (22.0%)	4 (16.0%)	
A bit	17 (19.5%)	4 (19.0%)	8 (19.5%)	5 (20.0%)	
Moderately	28 (32.2%)	9 (42.9%)	11 (26.8%)	8 (32.0%)	
A lot	18 (20.7%)	7 (33.3%)	6 (14.6%)	5 (20.0%)	
Absolutely	1 (1.15%)	0 (0.00%)	0 (0.00%)	1 (4.00%)	

**Table A6:** Prior use of online medical and non-medical services by the participants in the survey located elsewhere in the world.

Variable	Intention to Use Telemedicine in the Future				
	Overall (n = 114)	Intent (n = 28)	Undecided (n = 47)	No Intent (n = 39)	*p*-Value
Do you use the following online services? Banking services, Food purchases, Clothing purchases, Electrical product purchases, Hotel reservations, Vacation arrangements (planning, purchases), Health consulting, Government services (n = 114)					<0.0001
No	0 (0.00%)	0 (0.00%)	0 (0.00%)	0 (0.00%)	
Yes	114 (100%)	28 (100%)	47 (100%)	39 (100%)	
Do you use social media (reading, posting) for information and/or advice (not necessarily concerning a medical problem)? (n = 114)					<0.0001
Absolutely not	18 (15.8%)	2 (7.14%)	5 (10.6%)	11 (28.2%)	
A bit	20 (17.5%)	3 (10.7%)	11 (23.4%)	6 (15.4%)	
Moderately	42 (36.8%)	9 (32.1%)	20 (42.6%)	13 (33.3%)	
A lot	24 (21.1%)	10 (35.7%)	8 (17.0%)	6 (15.4%)	
Always	10 (8.77%)	4 (14.3%)	3 (6.38%)	3 (7.69%)	
Do you use online medical services? (n = 114)					0.003
No, not interested in using them	29 (25.4%)	2 (7.14%)	13 (27.7%)	14 (35.9%)	
No, but willing to experiment	30 (26.3%)	4 (14.3%)	17 (36.2%)	9 (23.1%)	
Yes, I used them for the first time during the COVID-19 pandemic.	27 (23.7%)	9 (32.1%)	7 (14.9%)	11 (28.2%)	
Yes, I used them before the COVID-19 pandemic.	28 (24.6%)	13 (46.4%)	10 (21.3%)	5 (12.8%)	
Before seeing a doctor, do you use social media to find answers to your question? (n = 114)					0.252
Absolutely not	33 (28.9%)	6 (21.4%)	14 (29.8%)	13 (33.3%)	
A bit	23 (20.2%)	4 (14.3%)	7 (14.9%)	12 (30.8%)	
Moderately	32 (28.1%)	11 (39.3%)	16 (34.0%)	5 (12.8%)	
A lot	12 (10.5%)	3 (10.7%)	4 (8.51%)	5 (12.8%)	
Always	14 (12.3%)	4 (14.3%)	6 (12.8%)	4 (10.3%)	
Has your knowledge of using other online services helped you use online medical services? (n = 114)					<0.0001
I have not tried online services.	28 (24.6%)	2 (7.14%)	11 (28.2%)	15 (31.9%)	
Absolutely not	8 (7.02%)	1 (3.57%)	5 (12.8%)	2 (4.26%)	
A bit	32 (28.1%)	9 (32.1%)	9 (23.1%)	14 (29.8%)	
Moderately	23 (20.2%)	6 (21.4%)	7 (17.9%)	10 (21.3%)	
A lot	12 (10.5%)	6 (21.4%)	4 (10.3%)	2 (4.26%)	
Absolutely	11 (9.65%)	4 (14.3%)	3 (7.69%)	4 (8.51%)	
