# Supplementary material for: Reasons for Utilizing Telemedicine during and after the COVID-19 Pandemic: An Internet-Based International Study
Source: J Clin Med. 2021 Nov 25;10(23):5519. doi: 10.3390/jcm10235519 (PMC8658517; doi:10.3390/jcm10235519)
Supplement: Supplementary file 1 [file jcm-10-05519-s001.zip › jcm-1464970-supplementary_AB_20211124/JCM_Reasons_telemedicine_COVID19_S1.pdf]

Table S1: Survey questionnaire

Survey questionnaire — English version
Telemedicine — Use of online medical technology
Dear participant,
As part of our graduation project in Industrial Engineering at the Holon Institute of Technology (HIT) in Israel, we are conducting a survey on the use of telemedicine services.
Our objective is to understand how remote consultation means are perceived in the context of the COVID-19 pandemic and thereafter.
Your answers to this questionnaire are voluntary, anonymous, and unpaid. This questionnaire has been approved by the Ethics Committee of the Faculty of Industrial Engineering and Technology Management of the Holon Institute of Technology (HIT).
The data collected will only be used in the context of this study and will not be transmitted to any other entity.
*By moving to the next page of this survey, I hereby certify that I am over eighteen (18) years of age and I agree to take part in it.
Thank you in advance for your participation!
The research team

1	Do you use the following online services?<br>(You can select multiple answers.)
	Banking services
	Food purchases
	Clothing purchases
	Electrical and electronic products purchases
	Hotel room reservations
	Vacation and travel reservations or purchases
	Health consulting services (experts in nutrition / dietetics, psychologists, etc.)
	Government administrative services (licensing, etc.)
2	Do you use social media (reading, posting) for information and / or advice (not necessarily concerning a medical problem)?
	Absolutely not
	A bit
	Moderately
	A lot
	Always
3	Do you use online medical services?
	No, I'm not interested in using them.
	No, but I'm willing to experiment.
	Yes, I used them before the COVID-19 pandemic.
	Yes, I used them for the first time during the COVID-19 pandemic.
4	Who around you uses online medical services?
	Grandparents
	Parents
	Family (partner / wife / husband, children)
	Friends
	Co-workers

	I don't know.
5	Has your knowledge of using other online services helped you use online medical services?
	I have not tried online services.
	Absolutely not
	A bit
	Moderately
	A lot
	Absolutely
6	What online services have you used / are you using?<br>(You can select multiple answers.)
	Appointment with a doctor, a nurse, a physiotherapist, or a dietitian
	Request for prescription(s) or renewal(s)
	Request sickness leave / a certificate of absence
	Referral to specialist physicians
	Obtaining the opinion of a specialist doctor (diagnosis)
	Consultation before surgery
	Remote consultation using a telemetry tool (example: Tyto)
	Obtaining nursing advice in case of emergency outside of consultations
	Remote emergency medicine (e.g., help with cardiac massage)
	Online purchase of pharmacy items, drugs, hygiene products, and cosmetics
	Obtaining / consulting the results of laboratory or imaging tests (examples: blood tests, smears, x-rays, etc.)
	None of the above cases
7	When booking an online appointment, can you choose a specific doctor?
	No
	Yes
	I don't know.
8	Before seeing a doctor, do you use social media to find answers to your question?
	Absolutely not
	A bit
	Moderately
	A lot
	Always
9	How often do you use a(n) (online) medical / health service?
	Never
	1–2 times a year
	1–2 times per half year
	1–2 times a month
	1–2 times a week
10	What type of service(s) do you prefer to use primarily with your doctor?
	Phone call
	Online video call
	Message using the "Write to doctor" feature
	Live chat
	No preference

11	After consulting a doctor online, you felt that you needed another face-to-face consultation (at the doctor's office).
	Have not had an online consultation with a doctor
	Absolutely disagree
	Do not agree
	Neutral
	Agree
	Totally agree
12	How satisfied are you with online medical services these days?
	Have not had an online consultation with a doctor
	Very dissatisfied
	Somewhat dissatisfied
	Neither satisfied nor dissatisfied
	Somewhat satisfied
	Very satisfied
13	What are the main factors that motivate you to use online medical services?<br>(Select up to 3 factors.)
	Doctor waiting time
	Fear of being with other (potentially sick) patients in the waiting room
	Ability to contact a doctor at any time (although this does not include a response)
	Receiving a medical answer anytime and anywhere in the world
	Saving time, without having to go to the clinic / practice and find parking
	Obtaining a prescription without having to go to the clinic / office
14	Are you aware of a device for online medical services* within your insurance fund?<br>* A unique external device for HMO members only (example: Tyto device)
	No
	Yes
	Do not wish to answer
15	Does the existence of online medical services devices affect your decision to switch from one health insurance fund to another?
	No
	Yes
	Do not wish to answer
16	Online medicine will come at the expense of a doctor's visit to the clinic.
	Absolutely disagree
	Do not agree
	Neutral
	Agree
	Totally agree
17	Following the COVID-19 pandemic, your perception of online medicine has changed.
	Absolutely disagree
	Do not agree
	Neutral
	Agree

	Totally agree
18	What factors have bothered you during an online consultation?<br>(Select up to 3 factors.)
	Interruption of the consultation without the possibility of renewing the call
	Unstable or incomprehensible (voice) communication
	Fear of being misunderstood and that the treatment will be of less quality compared to a face-to-face meeting.
	Fear of a response from a non-specialist doctor on a sent message or online chat
	The consultation did not take place (the healthcare professional did not call me).
	The doctor will not understand exactly how I am feeling and what my problem is.
	I am embarrassed to be filmed.
	The doctor cannot perform a basic physical examination (for example: it is not possible to understand through the camera how red the throat is).
	I cannot express myself well in writing if I use chat or a messaging system.
	Have not had an online consultation with a doctor
19	At the end of the COVID-19 pandemic crisis, I will preferably continue to use online services instead of going to the clinic / office.
	Absolutely disagree
	Do not agree
	Neutral
	Agree
	Totally agree
20	Online medical systems are accessible to the elderly.
	Absolutely disagree
	Do not agree
	Neutral
	Agree
	Totally agree
21	Have you met a senior who needed help using an online medical service? If so, what level of assistance was needed?
	I haven't met any.
	Only one general verbal explanation was needed.
	I helped with one of the steps (for example: making an appointment, logging in, using the application during the consultation, etc.).
	I supported the person throughout the process until its completion.
22	During the COVID-19 pandemic, you also asked for medical advice and / or treatment for issues that you had not addressed prior to this time.
	Absolutely disagree
	Do not agree
	Neutral
	Agree
	Totally agree
23	During the COVID-19 pandemic, you helped loved ones seek advice and / or medical treatment for issues they had not addressed before this time.
	Absolutely disagree
	Do not agree

	Neutral
	Agree
	Totally agree
A few more questions about you and we're done.	
24	What is your age?
	18–24
	25–34
	35–44
	45–54
	55–64
	65+
	Not interested in answering
25	What is your gender?
	Male
	Female
	Not interested in answering
26	What is your marital status?
	Single
	Married
	Divorced
	Widowed
	Not interested in answering
27	How many children do you have?
	0
	1–2
	3–4
	5+
	Not interested in answering
28	In what sort of area do you live?
	City
	Suburb or periphery
	Not interested in answering
29	Country of residence
	List of all the world countries in January 2021
30	City of residence
	Free text
31	Which health insurance plan or organization are you a member of?<br>(You don't have to answer if you are not interested.)
	Free text
32	We would be happy to receive comments and responses from you on the questionnaire.
	Free text

Thank you for your participation!
