# Supplementary material for: Reasons for Utilizing Telemedicine during and after the COVID-19 Pandemic: An Internet-Based International Study
Source: J Clin Med. 2021 Nov 25;10(23):5519. doi: 10.3390/jcm10235519 (PMC8658517; doi:10.3390/jcm10235519)
Supplement: Supplementary file 1 [file jcm-10-05519-s001.zip › jcm-1464970-supplementary_AB_20211124/JCM_Reasons_telemedicine_COVID19_S2.pdf]

Table S2: List of the countries of the participants' not located in Israel or in Uruguay

Country	Participants (n/N, %) from elsewhere (not Israel and not Uruguay)	Participants (n/N, %) from elsewhere in regard of the overall participants
Bahrain	1/114, 0.88	1/473, 0.21
Canada	2/114, 1.75	2/473, 0.42
Chile	2/114, 1.75	2/473, 0.42
Colombia	2/114, 1.75	2/473, 0.42
China	3/114, 2.63	3/473, 0.63
Denmark	2/114, 1.75	2/473, 0.42
France	39/114, 41.2	39/473, 9.94
Germany	7/114, 6.14	7/473, 1.48
India	1/114, 0.88	1/473, 0.21
Ireland	1/114, 0.88	1/473, 0.21
Italy	2/114, 1.75	2/473, 0.42
Romania	10/114, 8.77	10/473, 2.11
Russia	4/114, 3.51	4/473, 0.85
Singapore	1/114, 0.88	1/473, 0.21
Switzerland	4/114, 3.51	4/473, 0.85
United Kingdom	2/114, 1.75	2/473, 0.42
United States of America	24/114, 20.2	24/473, 4.86
