# Supplementary material for: Reasons for Utilizing Telemedicine during and after the COVID-19 Pandemic: An Internet-Based International Study
Source: J Clin Med. 2021 Nov 25;10(23):5519. doi: 10.3390/jcm10235519 (PMC8658517; doi:10.3390/jcm10235519)
Supplement: Supplementary file 1 [file jcm-10-05519-s001.zip › jcm-1464970-supplementary_AB_20211124/JCM_Reasons_telemedicine_COVID19_S3.pdf]

Table S3. *Health services consumption by the Israeli participants in the survey*

Variable	Intention to use telemedicine in the future				
	**Overall (n=272)**	**Agree (n=139)**	**Neutral (n=79)**	**Disagree (n=54)**	**p-Value**
**How often do you use a(n) (online) medical / health service? (n=272)**					<0.0001
Never	24 (8.82%)	1 (0.72%)	10 (12.7%)	13 (24.1%)	
1–2 times a year	46 (16.9%)	17 (12.2%)	15 (19.0%)	14 (25.9%)	
1–2 times per half year	100 (36.8%)	53 (38.1%)	29 (36.7%)	18 (33.3%)	
1–2 times a month	88 (32.4%)	56 (40.3%)	23 (29.1%)	9 (16.7%)	
1–2 times a week	14 (5.15%)	12 (8.63%)	2 (2.53%)	0 (0.00%)	
**What type of service(s) do you prefer to use primarily with your doctor? (n=272)**					0.007
Phone call	93 (34.2%)	38 (27.3%)	28 (35.4%)	27 (50.0%)	
Online video call	27 (9.93%)	19 (13.7%)	6 (7.59%)	2 (3.70%)	
Message using the "Write to doctor" feature	83 (30.5%)	51 (36.7%)	18 (22.8%)	14 (25.9%)	
Live chat	27 (9.93%)	16 (11.5%)	8 (10.1%)	3 (5.56%)	
No preference	42 (15.4%)	15 (10.8%)	19 (24.1%)	8 (14.8%)	
**When booking an online appointment, can you choose a specific doctor?(n=272)**					0.083
No	27 (9.93%)	13 (9.35%)	8 (10.1%)	6 (11.1%)	
Yes	192 (70.6%)	107 (77.0%)	48 (60.8%)	37 (68.5%)	
I don't know	53 (19.5%)	19 (13.7%)	23 (29.1%)	11 (20.4%)	
**Who around you uses online medical services?(n=272)**					
Family (grandparents, parents, partner / wife / husband, children)	163 (59.9%)	98 (70.5%)	46 (58.2%)	19 (35.2%)	<0.001
Friends	116 (42.6%)	70 (50.4%)	32 (40.5%)	14 (25.9%)	0.008
Coworkers	77 (28.3%)	53 (38.1%)	18 (22.8%)	6 (11.1%)	<0.001
I don't know	85 (31.2%)	30 (21.6%)	25 (31.6%)	30 (55.6%)	<0.001
**What are the main factors that motivate you to use online medical services? (Select up to 3 factors.) (n=272)**					
Doctor waiting time	126 (46.3%)	74 (53.2%)	32 (40.5%)	20 (37.0%)	0.060
Fear of being with other (potentially sick) patients in the waiting room	51 (18.8%)	32 (23.0%)	14 (17.7%)	5 (9.26%)	0.086
Ability to contact a doctor at any time (although this does not include a response)	85 (31.2%)	51 (36.7%)	26 (32.9%)	8 (14.8%)	0.012
Receiving a medical answer anytime and anywhere in the world	82 (30.1%)	49 (35.3%)	24 (30.4%)	9 (16.7%)	0.041
Saving time, without having to go to the clinic / practice and find parking	197 (72.4%)	115 (82.7%)	52 (65.8%)	30 (55.6%)	<0.001
Obtaining a prescription without having to go to the clinic / office	101 (37.1%)	38 (27.3%)	31 (39.2%)	32 (59.3%)	<0.001
**What online services have you used / are you using? (You can select multiple answers.) (n=272)**					
Appointment with a doctor, a nurse, a physiotherapist, or a dietitian	153 (56.2%)	94 (67.6%)	40 (50.6%)	19 (35.2%)	<0.001
Requesting prescription(s) or renewal(s)	215 (79.0%)	120 (86.3%)	62 (78.5%)	33 (61.1%)	0.001
Requesting sickness leave / certificate of absence	159 (58.5%)	93 (66.9%)	43 (54.4%)	23 (42.6%)	0.006
Referral to specialist physicians	147 (54.0%)	83 (59.7%)	40 (50.6%)	24 (44.4%)	0.124
Obtaining the opinion of a specialist doctor (diagnosis)	36 (13.2%)	22 (15.8%)	11 (13.9%)	3 (5.56%)	0.164
Consultation before surgery	5 (1.84%)	2 (1.44%)	3 (3.80%)	0 (0.00%)	0.341
Remote consultation using a telemetry tool (example: Tyto)	14 (5.15%)	13 (9.35%)	1 (1.27%)	0 (0.00%)	0.005
Obtaining nursing advice in case of emergency outside of consultations	22 (8.09%)	12 (8.63%)	8 (10.1%)	2 (3.70%)	0.387

Remote emergency medicine (e.g., help with cardiac massage)	1 (0.37%)	1 (0.72%)	0 (0.00%)	0 (0.00%)	1.000
Online purchase of pharmacy items, drugs, hygiene products, and cosmetics	65 (23.9%)	41 (29.5%)	18 (22.8%)	6 (11.1%)	0.026
Obtaining / consulting the results of laboratory or imaging tests (examples: blood tests, smears, x-rays, etc.)	135 (49.6%)	82 (59.0%)	35 (44.3%)	18 (33.3%)	0.003
None of the above cases	17 (6.25%)	3 (2.16%)	6 (7.59%)	8 (14.8%)	0.003
