# Supplementary material for: Reasons for Utilizing Telemedicine during and after the COVID-19 Pandemic: An Internet-Based International Study
Source: J Clin Med. 2021 Nov 25;10(23):5519. doi: 10.3390/jcm10235519 (PMC8658517; doi:10.3390/jcm10235519)
Supplement: Supplementary file 1 [file jcm-10-05519-s001.zip › jcm-1464970-supplementary_AB_20211124/JCM_Reasons_telemedicine_COVID19_S4.pdf]

Table S4. *Health services consumption by the Uruguayan participants in the survey*

Variable	Intention to use telemedicine in the future				
	**Overall (n=87)**	**Agree (n=21)**	**Neutral (n=25)**	**Disagree (n=41)**	**p-Value**
**How often do you use a(n) (online) medical / health service? (n=87)**					0.114
Never	14 (16.1%)	1 (4.76%)	3 (12.0%)	10 (24.4%)	
1–2 times a year	24 (27.6%)	3 (14.3%)	8 (32.0%)	13 (31.7%)	
1–2 times per half year	33 (37.9%)	11 (52.4%)	8 (32.0%)	14 (34.1%)	
1–2 times a month	14 (16.1%)	6 (28.6%)	5 (20.0%)	3 (7.32%)	
1–2 times a week	2 (2.30%)	0 (0.00%)	1 (4.00%)	1 (2.44%)	
**What type of service(s) do you prefer to use primarily with your doctor? (n=87)**					0.666
Phone call	42 (48.3%)	13 (61.9%)	9 (36.0%)	20 (48.8%)	
Online video call	25 (28.7%)	6 (28.6%)	9 (36.0%)	10 (24.4%)	
Message using the "Write to doctor" feature	2 (2.30%)	0 (0.00%)	1 (4.00%)	1 (2.44%)	
Live chat	1 (1.15%)	0 (0.00%)	0 (0.00%)	1 (2.44%)	
No preference	17 (19.5%)	2 (9.52%)	6 (24.0%)	9 (22.0%)	
**When booking an online appointment, can you choose a specific doctor? (n=87)**					0.550
No	6 (6.90%)	0 (0.00%)	2 (8.00%)	4 (9.76%)	
Yes	63 (72.4%)	18 (85.7%)	18 (72.0%)	27 (65.9%)	
I don't know	18 (20.7%)	3 (14.3%)	5 (20.0%)	10 (24.4%)	
**Who around you use online medical services? (n=87)**					
Family (grandparents, parents, partner / wife / husband, children)	45 (51.7%)	16 (76.2%)	17 (68.0%)	12 (29.3%)	<0.001
Friends	40 (46.0%)	13 (61.9%)	11 (44.0%)	16 (39.0%)	0.225
Coworkers	32 (36.8%)	10 (47.6%)	10 (40.0%)	12 (29.3%)	0.338
I don't know	25 (28.7%)	3 (14.3%)	5 (20.0%)	17 (41.5%)	0.043
**What are the main factors that motivate you to use online medical services? (Select up to 3 factors.) (n=87)**					
Doctor waiting time	26 (29.9%)	9 (42.9%)	11 (44.0%)	6 (14.6%)	0.013
Fear of being with other (potentially sick) patients in the waiting room	35 (40.2%)	11 (52.4%)	12 (48.0%)	12 (29.3%)	0.138
Ability to contact a doctor at any time (although this does not include a response)	27 (31.0%)	7 (33.3%)	6 (24.0%)	14 (34.1%)	0.665
Receiving a medical answer anytime and anywhere in the world	18 (20.7%)	7 (33.3%)	7 (28.0%)	4 (9.76%)	0.043
Saving time, without having to go to the clinic / practice and find parking	14 (16.1%)	8 (38.1%)	4 (16.0%)	2 (4.88%)	0.003
Obtaining a prescription without having to go to the clinic / office	53 (60.9%)	15 (71.4%)	15 (60.0%)	23 (56.1%)	0.501
**What online services have you used / are you using? (You can select multiple answers.) (n=87)**					
Appointment with a doctor, a nurse, a physiotherapist, or a dietitian	49 (56.3%)	17 (81.0%)	13 (52.0%)	19 (46.3%)	0.030
Requesting prescription(s) or renewal(s)	50 (57.5%)	17 (81.0%)	15 (60.0%)	18 (43.9%)	0.019
Requesting sickness leave / certificate of absence	7 (8.05%)	2 (9.52%)	2 (8.00%)	3 (7.32%)	1.000
Referral to specialist physicians	19 (21.8%)	7 (33.3%)	4 (16.0%)	8 (19.5%)	0.397
Obtaining the opinion of a specialist doctor (diagnosis)	22 (25.3%)	8 (38.1%)	8 (32.0%)	6 (14.6%)	0.087
Consultation before surgery	1 (1.15%)	0 (0.00%)	1 (4.00%)	0 (0.00%)	0.529
Remote consultation using a telemetry tool (example: Tyto)	0 (0.00%)	0 (0.00%)	0 (0.00%)	0 (0.00%)	.

Obtaining nursing advice in case of emergency outside of consultations	2 (2.30%)	1 (4.76%)	0 (0.00%)	1 (2.44%)	0.726
Remote emergency medicine (e.g., help with cardiac massage)	3 (3.45%)	0 (0.00%)	2 (8.00%)	1 (2.44%)	0.441
Online purchase of pharmacy items, drugs, hygiene products, and cosmetics	28 (32.2%)	12 (57.1%)	5 (20.0%)	11 (26.8%)	0.016
Obtaining / consulting the results of laboratory or imaging tests (examples: blood tests, smears, x-rays, etc.)	64 (73.6%)	19 (90.5%)	18 (72.0%)	27 (65.9%)	0.112
None of the above cases	8 (9.20%)	0 (0.00%)	2 (8.00%)	6 (14.6%)	0.189
