# Supplementary material for: Reasons for Utilizing Telemedicine during and after the COVID-19 Pandemic: An Internet-Based International Study
Source: J Clin Med. 2021 Nov 25;10(23):5519. doi: 10.3390/jcm10235519 (PMC8658517; doi:10.3390/jcm10235519)
Supplement: Supplementary file 1 [file jcm-10-05519-s001.zip › jcm-1464970-supplementary_AB_20211124/JCM_Reasons_telemedicine_COVID19_S5.pdf]

Table S5. *Health services consumption by the participants in the survey located elsewhere in the world*

Variable	Intention to use telemedicine in the future				
	**Overall (n=114)**	**Agree (n=28)**	**Neutral (n=47)**	**Disagree (n=39)**	**p-Value**
**How often do you use a(n) (online) medical / health service? (n=114)**					0.064
Never	39 (34.2%)	4 (14.3%)	19 (40.4%)	16 (41.0%)	
1–2 times a year	43 (37.7%)	10 (35.7%)	18 (38.3%)	15 (38.5%)	
1–2 times per half year	20 (17.5%)	7 (25.0%)	8 (17.0%)	5 (12.8%)	
1–2 times a month	11 (9.65%)	6 (21.4%)	2 (4.26%)	3 (7.69%)	
1–2 times a week	1 (0.88%)	1 (3.57%)	0 (0.00%)	0 (0.00%)	
**What type of service(s) do you prefer to use primarily with your doctor? (n=114)**					0.872
Phone call	28 (24.6%)	8 (28.6%)	11 (23.4%)	9 (23.1%)	
Online video call	34 (29.8%)	11 (39.3%)	13 (27.7%)	10 (25.6%)	
Message using the "Write to doctor" feature	6 (5.26%)	1 (3.57%)	3 (6.38%)	2 (5.13%)	
Live chat	8 (7.02%)	2 (7.14%)	4 (8.51%)	2 (5.13%)	
No preference	38 (33.3%)	6 (21.4%)	16 (34.0%)	16 (41.0%)	
**When booking an online appointment, can you choose a specific doctor? (n=114)**					0.720
No	16 (14.0%)	2 (7.14%)	8 (17.0%)	6 (15.4%)	
Yes	62 (54.4%)	18 (64.3%)	23 (48.9%)	21 (53.8%)	
I don't know	36 (31.6%)	8 (28.6%)	16 (34.0%)	12 (30.8%)	
**Who around you use online medical services? (n=114)**					
Family (grandparents, parents, partner / wife / husband, children)	47 (41.2%)	17 (60.7%)	15 (31.9%)	15 (38.5%)	0.045
Friends	35 (30.7%)	12 (42.9%)	12 (25.5%)	11 (28.2%)	0.266
Coworkers	23 (20.2%)	8 (28.6%)	6 (12.8%)	9 (23.1%)	0.220
I don't know	53 (46.5%)	8 (28.6%)	26 (55.3%)	19 (48.7%)	0.076
**What are the main factors that motivate you to use online medical services? (Select up to 3 factors.) (n=114)**					
Doctor waiting time	47 (41.2%)	11 (39.3%)	21 (44.7%)	15 (38.5%)	0.820
Fear of being with other (potentially sick) patients in the waiting room	21 (18.4%)	10 (35.7%)	6 (12.8%)	5 (12.8%)	0.025
Ability to contact a doctor at any time (although this does not include a response)	36 (31.6%)	7 (25.0%)	17 (36.2%)	12 (30.8%)	0.597
Receiving a medical answer anytime and anywhere in the world	45 (39.5%)	14 (50.0%)	21 (44.7%)	10 (25.6%)	0.084
Saving time, without having to go to the clinic / practice and find parking	77 (67.5%)	23 (82.1%)	31 (66.0%)	23 (59.0%)	0.130
Obtaining a prescription without having to go to the clinic / office	44 (38.6%)	13 (46.4%)	14 (29.8%)	17 (43.6%)	0.263
**What online services have you used / are you using? (You can select multiple answers.) (n=114)**					
Appointment with a doctor, a nurse, a physiotherapist, or a dietitian	74 (64.9%)	21 (75.0%)	31 (66.0%)	22 (56.4%)	0.285
Requesting prescription(s) or renewal(s)	40 (35.1%)	15 (53.6%)	13 (27.7%)	12 (30.8%)	0.059
Requesting sickness leave / certificate of absence	12 (10.5%)	6 (21.4%)	3 (6.38%)	3 (7.69%)	0.135
Referral to specialist physicians	18 (15.8%)	9 (32.1%)	3 (6.38%)	6 (15.4%)	0.018
Obtaining the opinion of a specialist doctor (diagnosis)	19 (16.7%)	10 (35.7%)	5 (10.6%)	4 (10.3%)	0.014
Consultation before surgery	6 (5.26%)	3 (10.7%)	1 (2.13%)	2 (5.13%)	0.276
Remote consultation using a telemetry tool (example: Tyto)	4 (3.51%)	2 (7.14%)	1 (2.13%)	1 (2.56%)	0.557
Obtaining nursing advice in case of emergency outside of consultations	4 (3.51%)	2 (7.14%)	1 (2.13%)	1 (2.56%)	0.557

Remote emergency medicine (e.g., help with cardiac massage)	2 (1.75%)	2 (7.14%)	0 (0.00%)	0 (0.00%)	0.059
Online purchase of pharmacy items, drugs, hygiene products, and cosmetics	27 (23.7%)	12 (42.9%)	7 (14.9%)	8 (20.5%)	0.019
Obtaining / consulting the results of laboratory or imaging tests (examples: blood tests, smears, x-rays, etc.)	37 (32.5%)	14 (50.0%)	15 (31.9%)	8 (20.5%)	0.039
None of the above cases	25 (21.9%)	1 (3.57%)	13 (27.7%)	11 (28.2%)	0.026
