# Supplementary material for: Reasons for Utilizing Telemedicine during and after the COVID-19 Pandemic: An Internet-Based International Study
Source: J Clin Med. 2021 Nov 25;10(23):5519. doi: 10.3390/jcm10235519 (PMC8658517; doi:10.3390/jcm10235519)
Supplement: Supplementary file 1 [file jcm-10-05519-s001.zip › jcm-1464970-supplementary_AB_20211124/JCM_Reasons_telemedicine_COVID19_S6.pdf]

Table S6. *Technology and communication as satisfaction triggers of the Israeli participants in the survey*

Variable	Intention to use telemedicine in the future				
	**Overall (n=272)**	**Agree (n=139)**	**Neutral (n=79)**	**Disagree (n=54)**	**p-Value**
**How satisfied are you with online medical services these days? (n=272)**					<0.0001
Have not had an online consultation with a doctor	49 (18.0%)	11 (7.91%)	18 (22.8%)	20 (37.0%)	
Very dissatisfied	6 (2.21%)	2 (1.44%)	1 (1.27%)	3 (5.56%)	
Somewhat dissatisfied	17 (6.25%)	7 (5.04%)	4 (5.06%)	6 (11.1%)	
Neither satisfied nor dissatisfied	109 (40.1%)	63 (45.3%)	31 (39.2%)	15 (27.8%)	
Somewhat satisfied	81 (29.8%)	47 (33.8%)	24 (30.4%)	10 (18.5%)	
Very satisfied	10 (3.68%)	9 (6.47%)	1 (1.27%)	0 (0.00%)	
**After consulting a doctor online, you felt that you needed another face-to-face consultation (at the doctor's office). (n=272)**					<0.001
Have not had an online consultation with a doctor	62 (22.8%)	18 (12.9%)	20 (25.3%)	24 (44.4%)	
Agree	47 (17.3%)	20 (14.4%)	11 (13.9%)	16 (29.6%)	
Neutral	77 (28.3%)	40 (28.8%)	33 (41.8%)	4 (7.41%)	
Disagree	86 (31.6%)	61 (43.9%)	15 (19.0%)	10 (18.5%)	
**What factors have bothered you during an online consultation? (Select up to 3 factors.) (n=272)**					
Interruption of the consultation without the possibility of renewing the call	50 (18.4%)	34 (24.5%)	12 (15.2%)	4 (7.41%)	0.016
Unstable or incomprehensible (voice) communication	27 (9.93%)	14 (10.1%)	9 (11.4%)	4 (7.41%)	0.750
Fear of being misunderstood and that the treatment will be of less quality compared to a face-to-face meeting	74 (27.2%)	30 (21.6%)	29 (36.7%)	15 (27.8%)	0.054
Fear of a response from a non-specialist doctor on a sent message or online chat	13 (4.78%)	7 (5.04%)	4 (5.06%)	2 (3.70%)	1.000
The consultation did not take place (the healthcare professional did not call me).	22 (8.09%)	16 (11.5%)	4 (5.06%)	2 (3.70%)	0.127
The doctor will not understand exactly how I am feeling and what my problem is.	66 (24.3%)	36 (25.9%)	19 (24.1%)	11 (20.4%)	0.723
I am embarrassed to be filmed.	16 (5.88%)	9 (6.47%)	6 (7.59%)	1 (1.85%)	0.337
The doctor cannot perform a basic physical examination (for example: it is not possible to understand through the camera how red the throat is).	131 (48.2%)	74 (53.2%)	39 (49.4%)	18 (33.3%)	0.044
I cannot express myself well in writing if I use chat or a messaging system.	26 (9.56%)	9 (6.47%)	10 (12.7%)	7 (13.0%)	0.209
Have not had an online consultation with a doctor	74 (27.2%)	25 (18.0%)	23 (29.1%)	26 (48.1%)	<0.001
**Have you met a senior who needed help using an online medical service? If so, what level of assistance was needed? (n=272)**					<0.001
I haven't met any.	144 (52.9%)	72 (51.8%)	41 (51.9%)	31 (57.4%)	
I helped with one of the steps (example: making an appointment, logging in, using the application during the consultation, etc.).	44 (16.2%)	21 (15.1%)	16 (20.3%)	7 (13.0%)	
Only one general verbal explanation was needed.	15 (5.51%)	8 (5.76%)	6 (7.59%)	1 (1.85%)	
Supported throughout the process until its completion	69 (25.4%)	38 (27.3%)	16 (20.3%)	15 (27.8%)	
**Are you aware of a device for online medical services* within your insurance fund? * A unique external device for HMO members only (example: Tyto device) (n=272)**					0.017
No	168 (61.8%)	77 (55.4%)	56 (70.9%)	35 (64.8%)	
Yes	95 (34.9%)	60 (43.2%)	19 (24.1%)	16 (29.6%)	
Do not wish to answer	9 (3.31%)	2 (1.44%)	4 (5.06%)	3 (5.56%)	
**Does the existence of online medical services devices affect your decision to switch from one health insurance fund to another? (n=272)**					0.507

No	216 (79.4%)	105 (75.5%)	67 (84.8%)	44 (81.5%)	
Yes	34 (12.5%)	19 (13.7%)	8 (10.1%)	7 (13.0%)	
Do not wish to answer	22 (8.09%)	15 (10.8%)	4 (5.06%)	3 (5.56%)	
**Following the COVID-19 pandemic, your perception of online medicine has changed. (n=272)**					<0.001
Agree	133 (48.9%)	79 (56.8%)	45 (57.0%)	9 (16.7%)	
Neutral	69 (25.4%)	25 (18.0%)	24 (30.4%)	20 (37.0%)	
Disagree	70 (25.7%)	35 (25.2%)	10 (12.7%)	25 (46.3%)	
**Online medicine will come at the expense of a doctor visit to the clinic. (n=272)**					<0.001
Agree	87 (32.0%)	69 (49.6%)	10 (12.7%)	8 (14.8%)	
Neutral	65 (23.9%)	29 (20.9%)	31 (39.2%)	5 (9.26%)	
Disagree	120 (44.1%)	41 (29.5%)	38 (48.1%)	41 (75.9%)	
**During the COVID-19 pandemic, you also asked for medical advice and / or treatment for issues that you had not addressed prior to this time. (n=272)**					0.001
Agree	99 (36.4%)	61 (43.9%)	25 (31.6%)	13 (24.1%)	
Neutral	78 (28.7%)	39 (28.1%)	29 (36.7%)	10 (18.5%)	
Disagree	95 (34.9%)	39 (28.1%)	25 (31.6%)	31 (57.4%)	
**During the COVID-19 pandemic, you helped loved ones seek advice and / or medical treatment for issues they had not addressed before this time. (n=272)**					0.002
Agree	128 (47.1%)	71 (51.1%)	40 (50.6%)	17 (31.5%)	
Neutral	76 (27.9%)	36 (25.9%)	27 (34.2%)	13 (24.1%)	
Disagree	68 (25.0%)	32 (23.0%)	12 (15.2%)	24 (44.4%)	
