# Supplementary material for: Reasons for Utilizing Telemedicine during and after the COVID-19 Pandemic: An Internet-Based International Study
Source: J Clin Med. 2021 Nov 25;10(23):5519. doi: 10.3390/jcm10235519 (PMC8658517; doi:10.3390/jcm10235519)
Supplement: Supplementary file 1 [file jcm-10-05519-s001.zip › jcm-1464970-supplementary_AB_20211124/JCM_Reasons_telemedicine_COVID19_S7.pdf]

Table S7. *Technology and communication as satisfaction triggers of the Uruguayan participants in the survey*

Variable	Intention to use telemedicine in the future				
	**Overall (n=87)**	**Agree (n=21)**	**Neutral (n=25)**	**Disagree (n=41)**	***p*-Value**
**How satisfied are you with online medical services these days? (n=87)**					0.008
Have not had an online consultation with a doctor	19 (21.8%)	2 (9.52%)	4 (16.0%)	13 (31.7%)	
Very dissatisfied	3 (3.45%)	0 (0.00%)	0 (0.00%)	3 (7.32%)	
Somewhat dissatisfied	3 (3.45%)	0 (0.00%)	0 (0.00%)	3 (7.32%)	
Neither satisfied nor dissatisfied	31 (35.6%)	7 (33.3%)	8 (32.0%)	16 (39.0%)	
Somewhat satisfied	30 (34.5%)	12 (57.1%)	12 (48.0%)	6 (14.6%)	
Very satisfied	1 (1.15%)	0 (0.00%)	1 (4.00%)	0 (0.00%)	
**After consulting a doctor online, you felt that you needed another face-to-face consultation (at the doctor's office). (n=87)**					0.051
Have not had an online consultation with a doctor	20 (23.0%)	1 (4.76%)	5 (20.0%)	14 (34.1%)	
Agree	33 (37.9%)	8 (38.1%)	8 (32.0%)	17 (41.5%)	
Neutral	15 (17.2%)	4 (19.0%)	7 (28.0%)	4 (9.76%)	
Disagree	19 (21.8%)	8 (38.1%)	5 (20.0%)	6 (14.6%)	
**What factors have bothered you during an online consultation? (Select up to 3 factors.) (n=87)**					
Interruption of the consultation without the possibility of renewing the call	5 (5.75%)	2 (9.52%)	1 (4.00%)	2 (4.88%)	0.709
Unstable or incomprehensible (voice) communication	6 (6.90%)	3 (14.3%)	0 (0.00%)	3 (7.32%)	0.125
Fear of being misunderstood and that the treatment will be of less quality compared to a face-to-face meeting	20 (23.0%)	5 (23.8%)	6 (24.0%)	9 (22.0%)	1.000
Fear of a response from a non-specialist doctor on a sent message or online chat	0 (0.00%)	0 (0.00%)	0 (0.00%)	0 (0.00%)	<0.0001
The consultation did not take place (the healthcare professional did not call me).	0 0.00%)	0 (0.00%)	0 (0.00%)	0 (0.00%)	<0.0001
The doctor will not understand exactly how I am feeling and what my problem is.	36 (41.4%)	7 (33.3%)	9 (36.0%)	20 (48.8%)	0.410
I am embarrassed to be filmed.	4 (4.60%)	1 (4.76%)	2 (8.00%)	1 (2.44%)	0.687
The doctor cannot perform a basic physical examination (for example: it is not possible to understand through the camera how red the throat is).	53 (60.9%)	12 (57.1%)	16 (64.0%)	25 (61.0%)	0.893
I cannot express myself well in writing if I use chat or a messaging system.	5 (5.75%)	0 (0.00%)	3 (12.0%)	2 (4.88%)	0.196
Have not had an online consultation with a doctor	18 (20.7%)	2 (9.52%)	4 (16.0%)	12 (29.3%)	0.181
**Have you met a senior who needed help using an online medical service? If so, what level of assistance was needed? (n=87)**					0.011
I haven't met any.	60 (69.0%)	11 (52.4%)	14 (56.0%)	35 (85.4%)	
I helped with one of the steps (example: making an appointment, logging in, using the application during the consultation, etc.).	12 (13.8%)	3 (14.3%)	7 (28.0%)	2 (4.88%)	
Only one general verbal explanation was needed.	2 (2.30%)	1 (4.76%)	1 (4.00%)	0 (0.00%)	
Supported throughout the process until its completion	13 (14.9%)	6 (28.6%)	3 (12.0%)	4 (9.76%)	
**Are you aware of a device for online medical services* within your insurance fund? * A unique external device for HMO members only (example: Tyto device) (n=87)**					<0.0001
No	75 (86.2%)	17 (81.0%)	23 (92.0%)	35 (85.4%)	0.785

Yes	11 (12.6%)	4 (19.0%)	2 (8.00%)	5 (12.2%)	
Do not wish to answer	1 (1.15%)	0 (0.00%)	0 (0.00%)	1 (2.44%)	
**Does the existence of online medical services devices affect your decision to switch from one health insurance fund to another? (n=87))**					0.367
No	54 (62.1%)	17 (81.0%)	14 (56.0%)	23 (56.1%)	
Yes	13 (14.9%)	2 (9.52%)	4 (16.0%)	7 (17.1%)	
Do not wish to answer	20 (23.0%)	2 (9.52%)	7 (28.0%)	11 (26.8%)	
**Following the COVID-19 pandemic, your perception of online medicine has changed. (n=87)**					<0.001
Agree	34 (39.1%)	15 (71.4%)	11 (44.0%)	8 (19.5%)	
Neutral	30 (34.5%)	5 (23.8%)	12 (48.0%)	13 (31.7%)	
Disagree	23 (26.4%)	1 (4.76%)	2 (8.00%)	20 (48.8%)	
**Online medicine will come at the expense of a doctor visit to the clinic. (n=87)**					0.001
Agree	7 (8.05%)	5 (23.8%)	1 (4.00%)	1 (2.44%)	
Neutral	13 (14.9%)	5 (23.8%)	6 (24.0%)	2 (4.88%)	
Disagree	67 (77.0%)	11 (52.4%)	18 (72.0%)	38 (92.7%)	
**During the COVID-19 pandemic, you also asked for medical advice and / or treatment for issues that you had not addressed prior to this time. (n=87)**					0.188
Agree	19 (21.8%)	6 (28.6%)	4 (16.0%)	9 (22.0%)	
Neutral	5 (5.75%)	0 (0.00%)	4 (16.0%)	1 (2.44%)	
Disagree	63 (72.4%)	15 (71.4%)	17 (68.0%)	31 (75.6%)	
**During the COVID-19 pandemic, you helped loved ones seek advice and / or medical treatment for issues they had not addressed before this time. (n=87)**					0.036
Agree	25 (28.7%)	11 (52.4%)	3 (12.0%)	11 (26.8%)	
Neutral	10 (11.5%)	1 (4.76%)	5 (20.0%)	4 (9.76%)	
Disagree	52 (59.8%)	9 (42.9%)	17 (68.0%)	26 (63.4%)	
