# Supplementary material for: Reasons for Utilizing Telemedicine during and after the COVID-19 Pandemic: An Internet-Based International Study
Source: J Clin Med. 2021 Nov 25;10(23):5519. doi: 10.3390/jcm10235519 (PMC8658517; doi:10.3390/jcm10235519)
Supplement: Supplementary file 1 [file jcm-10-05519-s001.zip › jcm-1464970-supplementary_AB_20211124/JCM_Reasons_telemedicine_COVID19_S8.pdf]

Table S8. *Technology and communication as satisfaction triggers of the participants in the survey located elsewhere in the world*

Variable	Intention to use telemedicine in the future				
	**Overall (n=114)**	**Agree (n=28)**	**Neutral (n=47)**	**Disagree (n=39)**	***p*-Value**
**How satisfied are you with online medical services these days? (n=114)**					<0.0001
Have not had an online consultation with a doctor	46 (40.4%)	4 (14.3%)	21 (44.7%)	21 (53.8%)	
Very dissatisfied	2 (1.75%)	1 (3.57%)	0 (0.00%)	1 (2.56%)	
Somewhat dissatisfied	10 (8.77%)	3 (10.7%)	2 (4.26%)	5 (12.8%)	
Neither satisfied nor dissatisfied	18 (15.8%)	2 (7.14%)	9 (19.1%)	7 (17.9%)	
Somewhat satisfied	33 (28.9%)	13 (46.4%)	15 (31.9%)	5 (12.8%)	
Very satisfied	5 (4.39%)	5 (17.9%)	0 (0.00%)	0 (0.00%)	
**After consulting a doctor online, you felt that you needed another face-to-face consultation (at the doctor's office). (n=114)**					0.013
Have not had an online consultation with a doctor	43 (37.7%)	6 (21.4%)	18 (38.3%)	19 (48.7%)	
Agree	26 (22.8%)	3 (10.7%)	12 (25.5%)	11 (28.2%)	
Neutral	24 (21.1%)	9 (32.1%)	8 (17.0%)	7 (17.9%)	
Disagree	21 (18.4%)	10 (35.7%)	9 (19.1%)	2 (5.13%)	
**What factors have bothered you during an online consultation? (Select up to 3 factors.) (n=114)**					
Interruption of the consultation without the possibility of renewing the call	11 (9.65%)	9 (32.1%)	1 (2.13%)	1 (2.56%)	<0.001
Unstable or incomprehensible (voice) communication	17 (14.9%)	6 (21.4%)	5 (10.6%)	6 (15.4%)	0.423
Fear of being misunderstood and that the treatment will be of less quality compared to a face-to-face meeting	24 (21.1%)	3 (10.7%)	8 (17.0%)	13 (33.3%)	0.055
Fear of a response from a non-specialist doctor on a sent message or online chat	9 (7.89%)	2 (7.14%)	3 (6.38%)	4 (10.3%)	0.905
The consultation did not take place (the healthcare professional did not call me).	5 (4.39%)	3 (10.7%)	0 (0.00%)	2 (5.13%)	0.059
The doctor will not understand exactly how I am feeling and what my problem is.	30 (26.3%)	10 (35.7%)	10 (21.3%)	10 (25.6%)	0.387
I am embarrassed to be filmed.	8 (7.02%)	4 (14.3%)	3 (6.38%)	1 (2.56%)	0.211
The doctor cannot perform a basic physical examination (for example: it is not possible to understand through the camera how red the throat is).	44 (38.6%)	8 (28.6%)	17 (36.2%)	19 (48.7%)	0.224
I cannot express myself well in writing if I use chat or a messaging system.	12 (10.5%)	3 (10.7%)	4 (8.51%)	5 (12.8%)	0.925
Have not had an online consultation with a doctor	43 (37.7%)	4 (14.3%)	21 (44.7%)	18 (46.2%)	0.013
**Have you met a senior who needed help using an online medical service? If so, what level of assistance was needed? (n=114)**					0.068
I haven't met any.	81 (71.1%)	15 (53.6%)	35 (74.5%)	31 (79.5%)	
I helped with one of the steps (example: making an appointment, logging in, using the application during the consultation, etc.).	15 (13.2%)	9 (32.1%)	4 (8.51%)	2 (5.13%)	
Only one general verbal explanation was needed.	4 (3.51%)	1 (3.57%)	2 (4.26%)	1 (2.56%)	
Supported throughout the process until its completion	14 (12.3%)	3 (10.7%)	6 (12.8%)	5 (12.8%)	
**Are you aware of a device for online medical services* within your insurance fund? * A unique external device for HMO members only (example: Tyto device) (n=114)**					0.657
No	94 (82.5%)	24 (85.7%)	37 (78.7%)	33 (84.6%)	

Yes	14 (12.3%)	4 (14.3%)	6 (12.8%)	4 (10.3%)	
Do not wish to answer	6 (5.26%)	0 (0.00%)	4 (8.51%)	2 (5.13%)	
**Does the existence of online medical services devices affect your decision to switch from one health insurance fund to another? (n=114)**					<0.001
No	74 (64.9%)	20 (71.4%)	21 (44.7%)	33 (84.6%)	
Yes	17 (14.9%)	6 (21.4%)	11 (23.4%)	0 (0.00%)	
Do not wish to answer	23 (20.2%)	2 (7.14%)	15 (31.9%)	6 (15.4%)	
**Following the COVID-19 pandemic, your perception of online medicine has changed. (n=114)**					0.278
Agree	44 (38.6%)	14 (50.0%)	18 (38.3%)	12 (30.8%)	
Neutral	38 (33.3%)	6 (21.4%)	19 (40.4%)	13 (33.3%)	
Disagree	32 (28.1%)	8 (28.6%)	10 (21.3%)	14 (35.9%)	
**Online medicine will come at the expense of a doctor visit to the clinic. (n=114)**					0.136
Agree	33 (28.9%)	9 (32.1%)	9 (19.1%)	15 (38.5%)	
Neutral	46 (40.4%)	12 (42.9%)	24 (51.1%)	10 (25.6%)	
Disagree	35 (30.7%)	7 (25.0%)	14 (29.8%)	14 (35.9%)	
**During the COVID-19 pandemic, you also asked for medical advice and / or treatment for issues that you had not addressed prior to this time. (n=114)**					0.162
Agree	29 (25.4%)	11 (39.3%)	8 (17.0%)	10 (25.6%)	
Neutral	46 (40.4%)	7 (25.0%)	24 (51.1%)	15 (38.5%)	
Disagree	39 (34.2%)	10 (35.7%)	15 (31.9%)	14 (35.9%)	
**During the COVID-19 pandemic, you helped loved ones seek advice and / or medical treatment for issues they had not addressed before this time. (n=114)**					0.548
Agree	35 (30.7%)	11 (39.3%)	15 (31.9%)	9 (23.1%)	
Neutral	34 (29.8%)	9 (32.1%)	12 (25.5%)	13 (33.3%)	
Disagree	45 (39.5%)	8 (28.6%)	20 (42.6%)	17 (43.6%)	
